# Tumor-secreted exosomal Wnt2B activates fibroblasts to promote cervical cancer progression

**DOI:** 10.1038/s41389-021-00319-w

**Published:** 2021-03-17

**Authors:** Luo-Jiao Liang, Yang Yang, Wen-Fei Wei, Xiang-Guang Wu, Rui-Ming Yan, Chen-Fei Zhou, Xiao-Jing Chen, Sha Wu, Wei Wang, Liang-Sheng Fan

**Affiliations:** 1 Department of Obstetrics and Gynecology, The First Affiliated Hospital of Guangzhou Medical University, Guangzhou, 510120 Guangdong China; 2 Department of Immunology, School of Basic Medical Sciences, Southern Medical University, Guangdong Provincial Key Laboratory of Proteomic, Guangzhou, 510515 Guangdong China

**Keywords:** Cervical cancer, Cancer microenvironment

## Abstract

The activation of stromal fibroblasts into cancer-associated fibroblasts (CAFs) has been suggested to promote primary tumor growth and progression; however, the mechanisms underlying the crosstalk between tumors and fibroblasts that drives stromal heterogeneity remain unknown. Here, we show that high Wnt2B levels were positively correlated with the number of CAFs in cervical cancer (CC). More importantly, Wnt2B was characteristically enriched in CC cell-secreted exosomes and transferred into fibroblasts to promote fibroblast activation via Wnt/β-catenin signaling, and inhibiting exosomal release or the Wnt/β-catenin signaling pathway diminished the activation induced by exosomal Wnt2B. Moreover, circulating exosomal Wnt2B also promoted CAF conversion in vitro and its expression was significantly higher in CC patients. In conclusion, our findings indicate that CC cell-derived Wnt2B can induce the activation of fibroblasts into CAFs, mainly via exosome-dependent secretion, thus providing directions for the development of diagnostic and therapeutic targets for CC progression.

## Introduction

It has recently been suggested that cancer progression is the result of evolving crosstalk between different cell types within the tumor microenvironment (TME)^1^. The remodeling of regional fibroblasts by cancer cells is associated with increased malignant progression and poor outcome in solid tumors such as cervical cancer (CC)^2^. Fibroblasts are a key cellular component of human tissues and tumors, and can be further divided into two types based on their response to their microenvironment: resting and activated fibroblasts^3^. Activated fibroblasts, which are characterized by α-smooth muscle actin (α-SMA) expression^4,5^, are transiently present during normal wound healing^6,7^ but persistently present in tumor tissues, where they are known as cancer-associated fibroblasts (CAFs)^8,9^. In addition to their lack of physiological role in homeostasis, extensive evidence suggests that CAFs are involved in stimulating cancer cell proliferation and progression^8,10^, confirming the biological and clinical significance of CAFs in the TME. However, the mechanisms via which normal fibroblasts (NFs) are transformed into CAFs by cancer cells remain largely unclear in CC.

Extracellular vesicles (EVs) are small membrane-enclosed particles that are released into the extracellular environment by multiple cell types, including cancer cells, which mediate intercellular communication by transporting signaling molecules as cargo^11,12^. EVs are markedly heterogeneous and are released from all cells in varying sizes and with different contents^11^. Nanoparticle tracking analysis data in our previous study indicated that CC cells release similar numbers of small EVs (also called exosome, 30–100 nm) with similar size distributions^13,14^. It has been shown that tumor cell-derived exosomes participate in the remodeling of the TME by delivering proteins, lipids, and nucleic acids^15^; however, the ability of these exosomes to modulate the phenotype and function of fibroblasts requires further investigation.

The recent discovery of the Wnt protein family and their presence in the extracellular environment indicates a potential role for these regulatory molecules in mediating stromal–epithelial crosstalk^16^. Since Wnt proteins are hydrophobic, they display different active extracellular forms that may influence their intercellular transport mechanisms^17^. Previous studies have demonstrated that a subset of extracellular Wnt proteins are co-localized with several exosomal markers in vivo, suggesting that Wnt proteins could be transported via exosomes^18,19^. Wnt2B, a member of the Wnt protein family, is upregulated as an oncogene in melanoma and nasopharyngeal carcinoma^20,21^. The loss of endogenous Wnt2B in fibroblasts increases the expression of senescence markers^21^, indicating that Wnt2B enhances fibroblast survival and differentiation. Therefore, we hypothesized that Wnt2B may influence CAF accumulation in the TME via exosomal secretion.

In this study, we investigated the mechanisms that underpin the differential activation of CAFs during cervical carcinogenesis and determined the relevance of these observations to CC. Our findings suggest that exosomal Wnt2B secreted by CC cells plays a crucial role in modifying stromal–epithelial crosstalk and in disrupting homeostasis in the TME, thus revealing a new target for abrogating CC progression.

## Results

### Wnt2B expression is positively associated with CAF abundance during cervical carcinogenesis

**Fig. 1:**
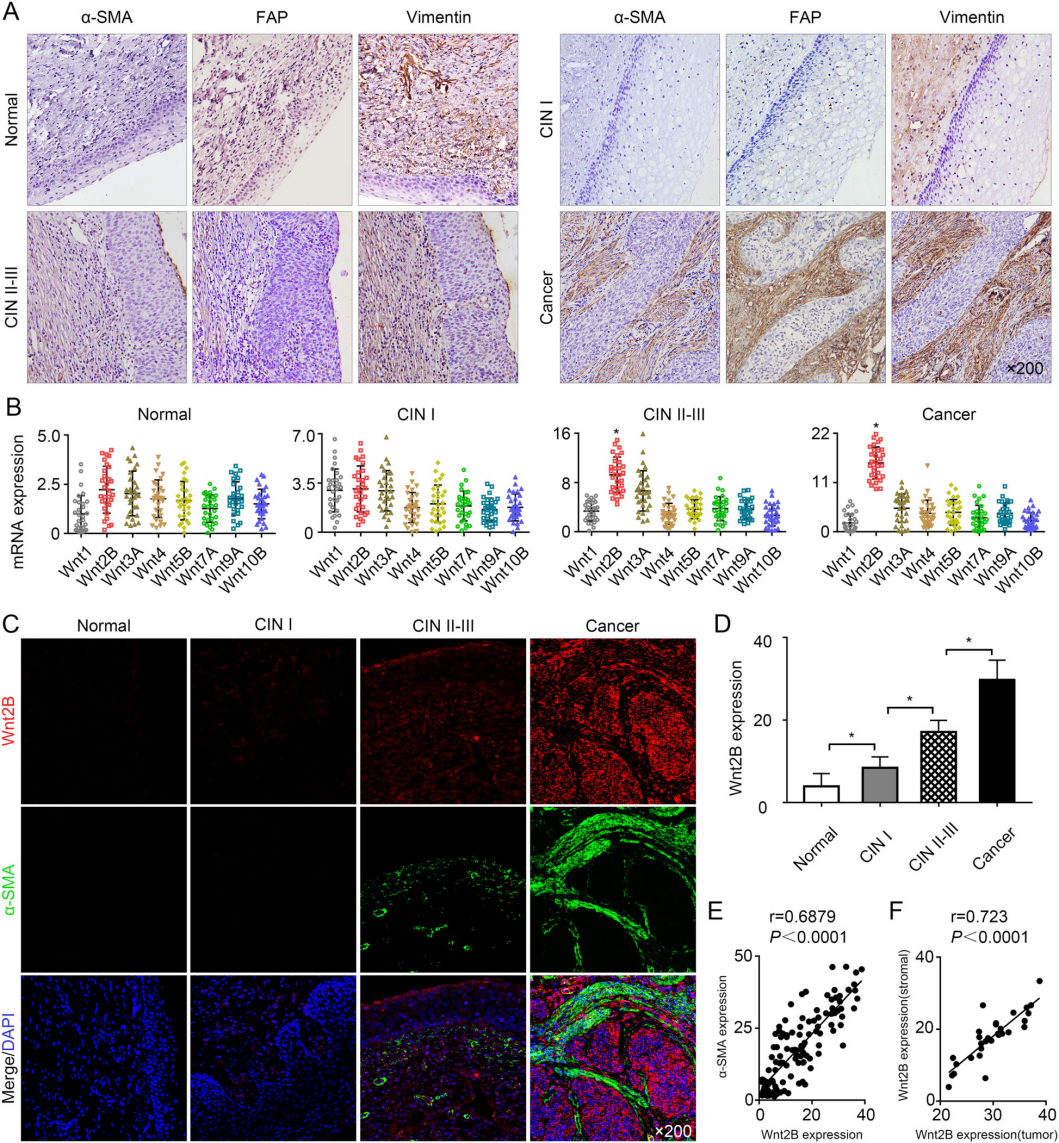
Wnt2B upregulation correlates with increased CAF numbers during cervical carcinogenesis. **A** α-SMA, FAP, and Vimentin immunostaining of cervical tissue samples (*n* = 10 normal cervix; *n* = 17 CIN I; *n* = 32 CIN II–III; *n* = 32 CC) at ×200 magnification. **B** Quantitative PCR analysis for Wnt expression in the four groups (*n* = 30 per group). **C** Cervical specimens were stained with Wnt2B (red), α-SMA (green), and DAPI (blue) for confocal microscopy at ×200 magnification. **D** Wnt2B expression levels were analyzed in the four groups. **E** Correlation of Wnt2B and α-SMA expression with CAF accumulation. **F** Correlation of Wnt2B expression between tumor and stromal. Error bars represent the mean ± SD of three independent experiments. *P < 0.05.

To evaluate CAF accumulation during cervical carcinogenesis, we detected α-SMA (CAF marker), fibroblast activation protein (FAP) (CAF marker), and Vimentin (fibroblast maker) levels in different grades of cervical dysplasia and CC. We found that the expression of α-SMA and FAP was significantly higher in cervical intraepithelial neoplasia II–III (CIN II–III) (*n* = 32) and CC (*n* = 32) samples than in normal cervix (*n* = 11) and CIN I lesion (*n* = 17) samples (*P* < 0.05, Fig. 1A and Table 1). However, the expression levels of stroma Vimentin did not significantly differ between any of the cervical specimens, suggesting a direct relationship between the malignancy of the grade of cervical lesions and the number of CAFs.

**Table 1:** Correlation of α-SMA or FAP expression with carcinoma progression.

Group	No.	α-SMA		High percentage (%)	*χ*^2^ (*P*)	FAP		High percentage (%)	*χ*^2^ (*P*)
		Low	High			Low	High		
Normal	11	10	1	9.09	38.04 (<0.001)	11	0	0	52.56 (<0.001)
CIN I	17	13	4	23.53		16	1	5.88	
CIN II–III	32	10	22	68.75		14	18	56.25	
Cancer	32	2	30	93.75		1	31	96.88	

Data are given as the number. Fisher’s exact test, *P* < 0.05.

*CIN* cervical intraepithelial neoplasia, *no.* number.

Several recent studies have reported that Wnt proteins regulate diverse cellular processes, including cell proliferation, survival, and cell fate specification^21,22^. Therefore, we investigated whether Wnt proteins are involved in the generation of tumor-promoting CAFs. Since Wnt2B expression gradually increased during cervical carcinogenesis and was positively correlated with high-risk HPV infection, but not significantly associated with age (Fig. 1B–D and Table S1), we further investigated the relationship between Wnt2B expression and CAF density. As shown in Fig. 1C, E, increased α-SMA expression was positively correlated with increased tumor Wnt2B expression (*r* = 0.6879, *P* < 0.0001). There are also high levels of Wnt2B in fibroblasts adjacent to the tumor; statistical analysis revealed that the expression of Wnt2B expression in tumor and in stromal is positively correlated (*r* = 0.723, *P* < 0.0001) (Fig. 1F), indicating that the transfer of tumor-derived Wnt2B to stroma may contribute towards CAF accumulation in CC.

### Wnt2B is highly enriched in exosomes secreted by CC cells

**Fig. 2:**
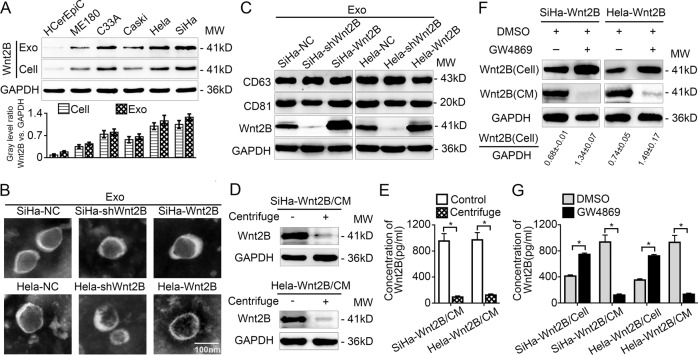
Wnt2B is highly enriched in exosomes secreted by CC cells. **A** Western blot analysis for Wnt2B in HCerEpiC, ME180, C33A, Caski, Hela, and SiHa cells and their corresponding exosomes. **B** Morphology of exosomes secreted by SiHa and Hela cells confirmed using transmission electron microscopy. Scale bar, 100 nm. **C** Western blot analysis for positive markers (CD63 and CD81) and Wnt2B expression in exosomes secreted by SiHa and Hela cells. **D**, **E** Western blot and ELISA analyses for Wnt2B in original CM from SiHa-Wnt2B or Hela-Wnt2B cells, or after overnight ultracentrifugation (160,000 × *g*). **F**, **G** Western blot and ELISA analyses for Wnt2B in the cell lysates and CM of SiHa-Wnt2B or Hela-Wnt2B treated with DMSO or GW4869 (15 μM) for 2 days. **A**, **C**, **D**, **F** Exosomes or CM secreted by the same number of cells; glyceraldehyde 3-phosphate dehydrogenase (GAPDH) was used as the loading control. Error bars represent the mean ± SD of three independent experiments. *P < 0.05.

Recent studies have shown that Wnt proteins are packaged into exosomes for functional release^12,18,19^. Therefore, we analyzed the Wnt2B secretory pathway in CC cells. The human cervical epithelial cell line (HCerEpiC) was chosen as a negative control for Wnt2B analysis. Western blot results for cell lysates and exosomes revealed that all CC cell lines showed higher expression of Wnt2B as compared to the HCerEpiC cell line. Moreover, Wnt2B was present in the exosomal fraction and more enriched in exosomes secreted by CC cells than in cell lysates (Fig. 2A). We also successfully transfected lenti-CD63-GFP-transfected SiHa and Hela cells with mCherry-tagged lentiviral vectors with Wnt2B expression and Wnt2B silencing, or a negative control, as confirmed by confocal microscopy (Supplementary Fig. 1A). Western blot and quantitative real-time PCR (qRT-PCR) analyses revealed that SiHa-Wnt2B/Hela-Wnt2B cells displayed significantly higher Wnt2B expression as compared to SiHa-NC/Hela-NC or SiHa-shWnt2B/Hela-shWnt2B cells (Supplementary Fig. 1B, C). In addition, since the Wnt2B expression level in ME180 was lower than that in other CC cells, we also established a Wnt2B-overexpressing ME180 cell line (ME180-Wnt2B) for further investigation (Supplementary Fig. 1A, D, E). The morphology of exosomes secreted by CC cells was confirmed using transmission electron microscopy (TEM), as a discoid vesicle-like structure with a diameter of 30–100 nm (Fig. 2B and Supplementary Fig. 2A). Wnt2B and exosomal marker (CD63 and CD81) detection confirmed that exosomes were isolated from SiHa-Wnt2B/Hela-Wnt2B/ME180-Wnt2B cells overexpressing Wnt2B (Fig. 2C and Supplementary Fig. 2B).

To investigate whether Wnt2B is mainly present in exosomes, we determined Wnt2B levels in the conditioned medium (CM) of SiHa-Wnt2B/Hela-Wnt2B/ME180-Wnt2B cells using enzyme-linked immunosorbent assays (ELISA) and western blotting. Wnt2B levels were lower in the CM of SiHa-Wnt2B/Hela-Wnt2B/ME180-Wnt2B cells after overnight ultracentrifugation (160,000 × *g*) than in the control groups (Fig. 2D, E and Supplementary Fig. 2C, D). Moreover, treating SiHa-Wnt2B/Hela-Wnt2B/ME180-Wnt2B cells with GW4869, an N-SMase inhibitor that blocks exosome secretion, increased intracellular Wnt2B levels, but significantly decreased CM Wnt2B levels as compared to that of the dimethylsulfoxide (DMSO) group (Fig. 2F, G and Supplementary Fig. 2E, F), suggesting that Wnt2B is mainly present in exosomes secreted by CC cells.

### Wnt2B can be transferred to fibroblasts via exosomes secreted by CC cells

**Fig. 3:**
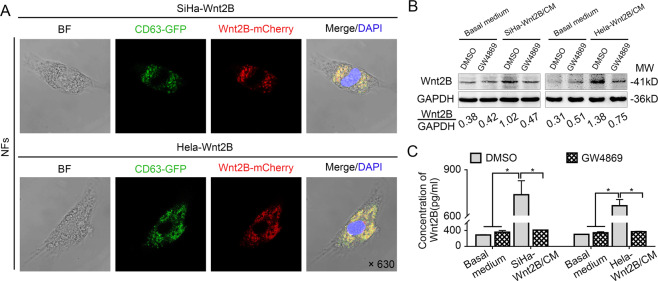
Wnt2B can be transferred to fibroblasts via exosome secretion. **A** Fibroblasts co-cultured with SiHa-Wnt2B and Hela-Wnt2B cells for 48 h imaged using confocal microscopy at ×630 magnification with morphology (bright field, BF), Wnt2B-mCherry (red), CD63-GFP (green), and DAPI (blue). **B** Western blot analysis for Wnt2B in fibroblasts cultured in the CM of SiHa-Wnt2B and Hela-Wnt2B cells after DMSO or GW4869 (15 μM) treatment for 2 days. GAPDH was used as the loading control. **C** ELISA analysis for Wnt2B in treated fibroblasts. Error bars represent the mean ± SD of three independent experiments. *P < 0.05.

To evaluate the transfer of exosomes between CC cells and fibroblasts, we isolated NFs from normal cervical samples and analyzed them using western blotting. The primary NFs showed positive Vimentin expression but negative α-SMA, FAP, CK (epithelial cell marker), and CD31 (endothelial cell marker) expression (Supplementary Fig. 3). After coculture with SiHa-Wnt2B/Hela-Wnt2B/ME180-Wnt2B cells for 48 h, confocal imaging revealed that green (labeled exosomes) and mCherry (labeled Wnt2B) fluorescent spots were co-localized in recipient NFs (Fig. 3A and Supplementary Fig. 4A). Moreover, Wnt2B expression was higher in NFs incubated with the CM of SiHa-Wnt2B/Hela-Wnt2B/ME180-Wnt2B cells than in those pre-treated with GW4869 (Fig. 3B, C and Supplementary Fig. 4B, C). However, Wnt2B expression was not upregulated in SiHa, Hela, and ME180 cells incubated with exosomes derived from NFs or CAFs (Supplementary Fig. 4D). These results indicate that Wnt2B could undergo horizontal transfer from CC cells to NFs via exosomes.

### Exosomal Wnt2B promotes fibroblast activation in vitro and in vivo

**Fig. 4:**
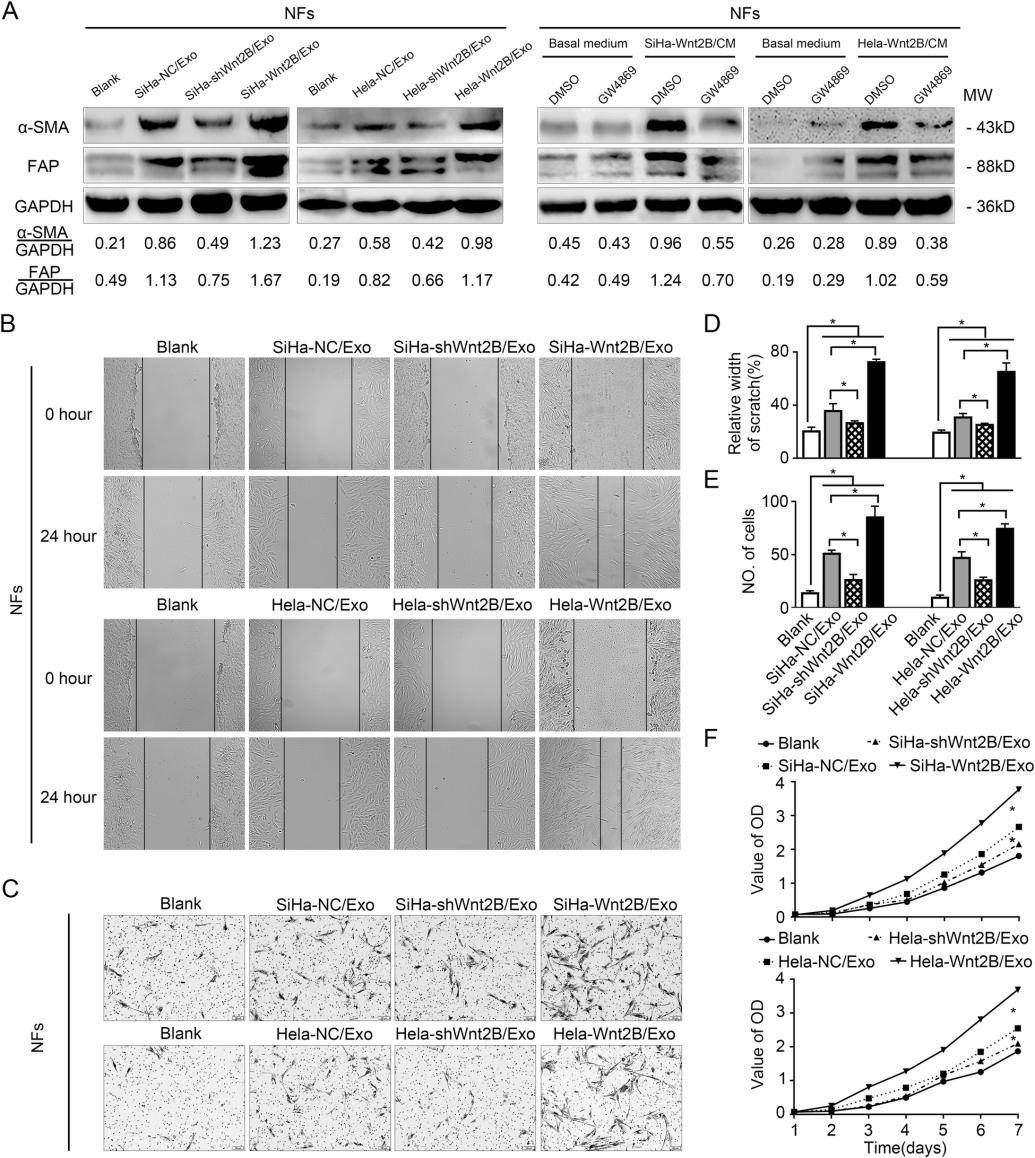
Exosomal Wnt2B promotes fibroblast activation. **A** Western blot analysis for α-SMA and FAP expression in NFs cultured alone (blank group) or co-cultured with the indicated exosomes (left). Western blot analysis for α-SMA and FAP expression in NFs pre-treated with CM from SiHa-Wnt2B and Hela-Wnt2B cells treated with DMSO or GW4869, while NFs treated with DMSO or GW4869 in the basal medium were used as the control group (right). **B** Wound healing assay using treated fibroblasts. **C** Migration assay using treated fibroblasts. **D** Cell migration quantified as a percentage of the wound-healed area. **E** Average number of invading cells per field from three independent experiments. **F** Proliferation assay using treated fibroblasts. Error bars represent the mean ± SD of three independent experiments. *P < 0.05.

To investigate the biological role of exosomal Wnt2B in fibroblast activation, we incubated NFs with exosomes secreted by CC cells for 48 h and analyzed the expression of α-SMA and FAP, while untreated NFs were used as the blank group. Western blot assays revealed that the expression levels of α-SMA and FAP were significantly higher in NFs treated with exosomes secreted by CC cells containing Wnt2B than those in other groups (Fig. 4A, left panel and Supplementary Fig. 5A, left panel). Consistently, blocking exosome secretion using GW4869 was found to suppress the upregulation of CAF markers induced by the CM of SiHa-Wnt2B/Hela-Wnt2B/ME180-Wnt2B cells (Fig. 4A, right panel and Supplementary Fig. 5A, right panel). Since activated fibroblasts may acquire synthetic functions that amplify their migration, recruitment, and proliferation, we assessed their activation state using wound healing, Transwell, and CCK8 assays. Increased migration and proliferation were observed in NFs treated with CM or exosomes containing Wnt2B (Fig. 4B–F and Supplementary Fig. 5B, C, F), while GW4869 alleviated these effects (Supplementary Fig. 5D, E, G).

To assess the ability of exosomes derived from Wnt2B-overexpressing CC cells to promote fibroblast activation in vivo, SiHa-Wnt2B/Hela-Wnt2B/ME180-Wnt2B were subcutaneously co-injected with fibroblasts into female nude mice. After tumor implantation, mice were intraperitoneally injected with GW4869 (2.0 mg/kg) or DMSO (solvent of GW4869, as a control), once every 2 days until 3 weeks. As shown in Supplementary Fig. 5H, compared with DMSO-treated groups, blocking exosome secretion with GW4869 shows a significantly decreased infiltration of activated fibroblasts, which is equivalent to that observed in vitro. Taken together, these results suggest that exosomal Wnt2B is a novel and highly potent regulator secreted by CC cells that could drive the conversion of NFs into CAFs.

### Exosomal Wnt2B activates Wnt/β-catenin signaling in fibroblasts

**Fig. 5:**
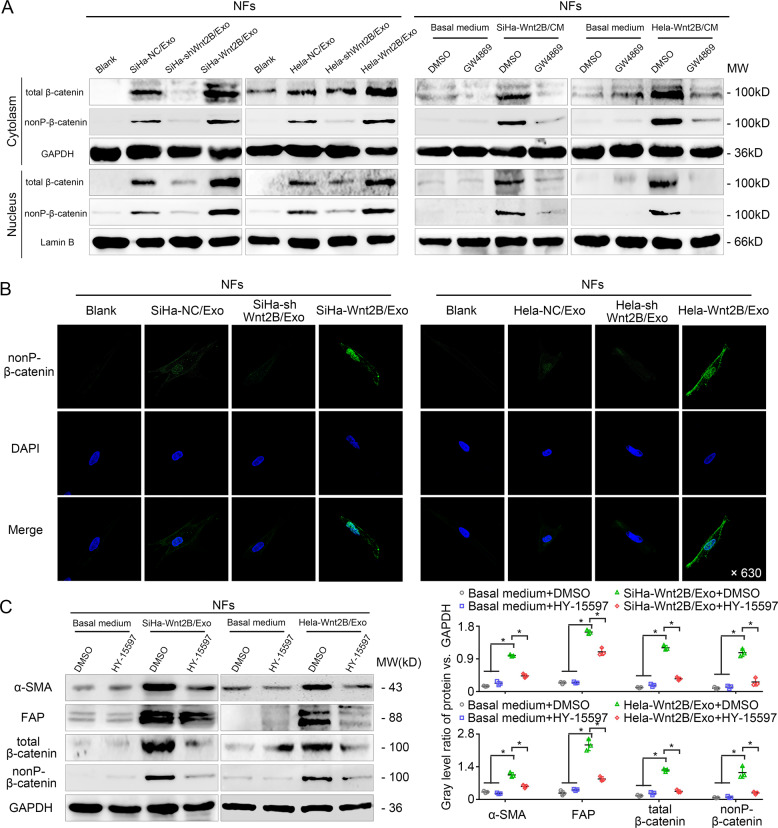
Wnt2B-driven CAF conversion depends on Wnt/β-catenin signaling. **A** Western blot analysis for Wnt/β-catenin signaling activation by total and non-phosphorylated (nonP) β-catenin (Ser33/37/Thr41) expression and localization in NFs cultured alone (blank group) or co-cultured with the exosomes (left). Western blot analysis for activation of Wnt/β-catenin signaling by total and nonP-β-catenin (Ser33/37/Thr41) expression and localization in NFs pre-treated with CM from SiHa-Wnt2B and Hela-Wnt2B cells treated with DMSO or GW4869, while NFs treated with DMSO or GW4869 in the basal medium were used as the control group (right). GAPDH was used as a loading control for cytoplasmic proteins, and laminin B as a loading control for nuclear proteins. **B** Immunofluorescence analysis of nonP-β-catenin (Ser33/37/Thr41) (green) localization in fibroblasts treated as indicated. Representative images are shown at ×630 magnification. **C** Immunoblot assays for CAF markers, total and nonP-β-catenin (Ser33/37/Thr41), in fibroblasts treated with exosomes and incubated with DMSO or HY-15597 (10 μM) for 1 day. Right: line graph showing the changes (indicated in gray) in the ratio of proteins to GAPDH described on the left. Error bars represent the mean ± SD of three independent experiments. *P < 0.05.

Since elevated Wnt signaling has been found to play an important role in the maintenance of CAF-like phenotypes^21,23^, we investigated whether exosomal Wnt2B secreted by CC cells is involved in fibroblast conversion by activating the Wnt/β-catenin signaling pathway. Unlike exosomes from NC and SiHa-shWnt2B/Hela-shWnt2B cells, those secreted by SiHa-Wnt2B/Hela-Wnt2B cells significantly increased nuclear accumulation of total, and in particular, non-phosphorylated (nonP) β-catenin levels in fibroblasts (Fig. 5A, B). Consistent with these results, GW4869 treatment abrogated the Wnt/β-catenin pathway activation induced by the CM of SiHa-Wnt2B/Hela-Wnt2B cells (Fig. 5A and Supplementary Fig. 6A). The stabilization and nuclear translocation of β-catenin promote transcription through a lymphoid enhancer factor/T cell factor (LEF/TCF) reporter construct. In the TCF-luciferase reporter assays employing the TOP-/FOP-flash system, the exosomes contained with Wnt2B significantly increased the activity of the TOP-flash reporter by ~4-fold compared with those in other groups (Supplementary Fig. 6B). Furthermore, GW4869 decreased relative TOP-flash reporter activity induced by the CM of SiHa-Wnt2B/Hela-Wnt2B/ME180-Wnt2B cells (Supplementary Fig. 6C). These results indicate that exosomal Wnt2B activates Wnt/β-catenin signaling in vitro. We also treated fibroblasts continuously with HY-15597, a potent Wnt signaling inhibitor, for 24 h before exosome treatment. Western blot analysis confirmed that HY-15597 treatment reduced the expression of α-SMA, FAP, and total and nonP-β-catenin in fibroblasts, indicating that activation of Wnt/β-catenin signaling is necessary for exosomal Wnt2B to induce CAF conversion (Fig. 5C). The above results were confirmed in ME180-NC/ME180-Wnt2B cells (Supplementary Fig. 7). Collectively, these findings suggest that exosomal Wnt2B promotes fibroblast activation via the Wnt/β-catenin pathway.

### Circulating exosomal Wnt2B is associated with fibroblast activation

**Fig. 6:**
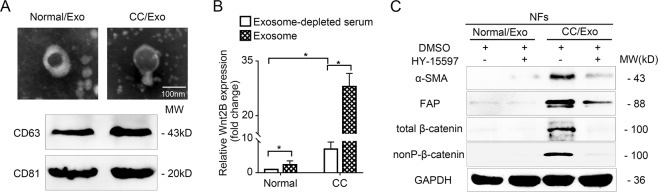
Circulating exosomal Wnt2B is associated with fibroblasts acquiring an activated phenotype. **A** Morphology of serum exosomes confirmed using transmission electron microscopy. Scale bar, 100 nm. Western blot analysis for exosomal markers (CD63 and CD81). **B** ELISA analysis for Wnt2B levels in circulating exosomes from healthy individuals (*n* = 4) and patients with CC (*n* = 8). **C** Western blot analysis for α-SMA, FAP, and total and nonP-β-catenin (Ser33/37/Thr41) in NFs treated with DMSO or HY-15597 before treatment with the two groups of exosomes. GAPDH was used as the loading control. Error bars represent the mean ± SD of three independent experiments. *P < 0.05.

To investigate whether Wnt2B secreted by cancer cells is present in the serum of patients with CC, we isolated and characterized exosomes from the serum samples of patients with CC (FIGO 2018, stage I–II, *n* = 8) and from healthy controls (*n* = 4). The typical morphology and size range of the exosomes purified from the serum samples were consistent with those purified from the culture supernatant (Fig. 6A); therefore, we examined exosomal Wnt2B levels in the serum samples. Wnt2B was mainly enriched in serum exosomes, while higher serum exosomal Wnt2B levels were detected in patients with CC than in the healthy controls (Fig. 6B). To investigate whether serum exosomal Wnt2B from patients with CC can functionally affect CAF conversion, we treated NFs with circulating exosomes obtained from patients with CC or healthy controls and measured the expression levels of α-SMA, FAP, and total and nonP-β-catenin. Circulating exosomes derived from patients with CC showed increased expression levels of α-SMA, FAP, and total and nonP-β-catenin in NFs, while HY-15597 attenuated this effect (Fig. 6C). These results indicate that circulating exosomal Wnt2B is associated with CAF activation, which was consistent with the observations in CC cell lines.

### Exosomal Wnt2B-activated CAFs promote tumor growth in vivo

**Fig. 7:**
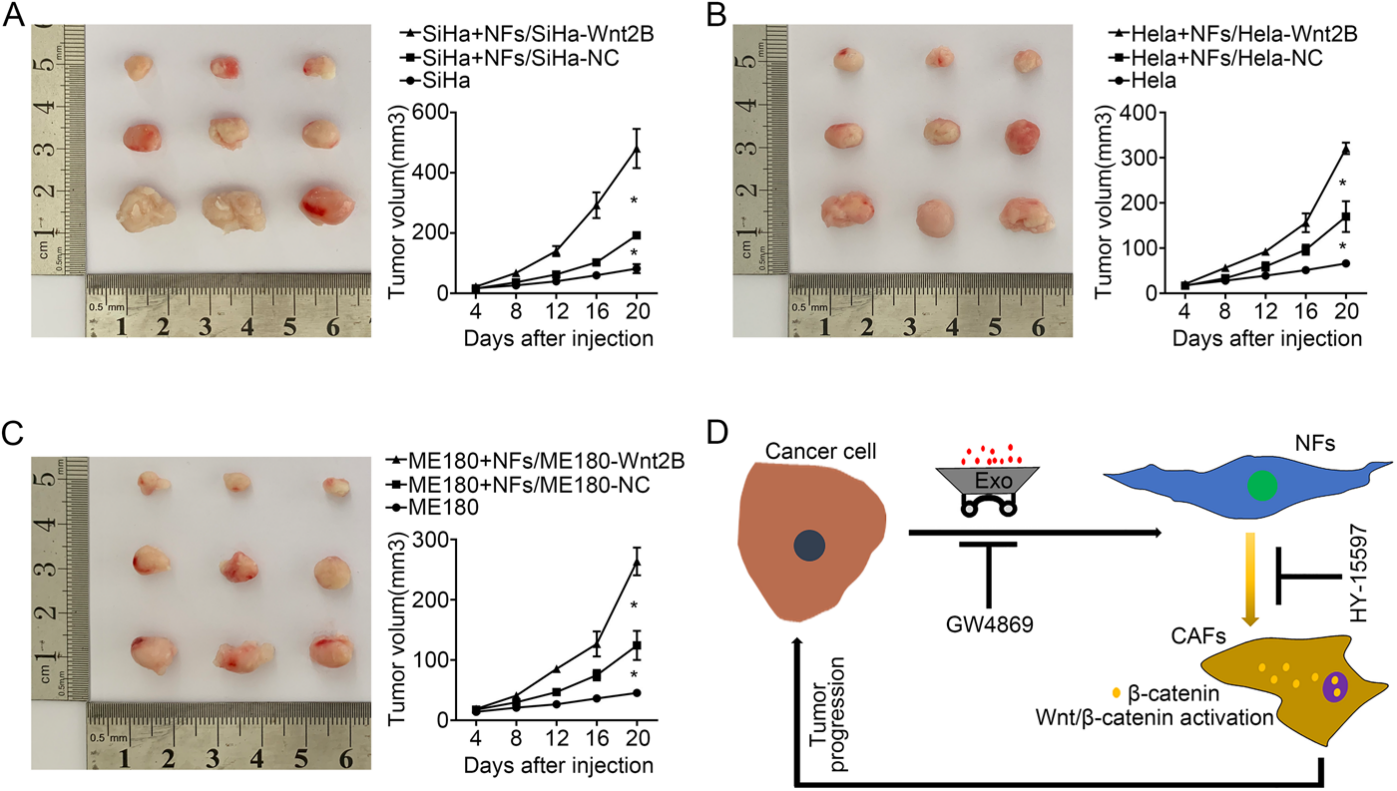
Exosomal Wnt2B-activated CAFs promote tumor growth in vivo. **A**–**C** The tumor volumes were evaluated in nude mice co-injected with CC cells and NFs pre-treated by SiHa-Wnt2B/Hela-Wnt2B/ME180-Wnt2B or by SiHa-NC/Hela-NC/ME180-NC. The control group only injected CC cells. Data are presented as the mean ± SD (*n* = 3; *P < 0.05). **D** Descriptive model showing the mechanism by which the exosomal Wnt2B secreted by CC cells activates CAFs through Wnt/β-catenin signaling, which subsequently promotes the development of cancer.

In light of our findings, exosomal Wnt2B promotes an altered stromal compartment in the tumor, we next characterized the functional properties of exosomal Wnt2B-activated CAFs. Subcutaneous model revealed that tumor growth was significantly increased in mice co-injected with CC cells and NFs pre-treated by SiHa-Wnt2B/Hela-Wnt2B/ME180-Wnt2B compared with SiHa-NC/Hela-NC/ME180-NC pre-treated groups or the control groups. These data confirmed that the fibroblasts activated by exosomal Wnt2B play an important role in CC progression (Fig. 7A–C).

Collectively, we propose a model to summarize our observations in the study in Fig. 7D. Exosomal Wnt2B secreted by CC cells promotes CAF conversion by activating the Wnt/β-catenin signaling, which further contributes to stroma remodeling and cancer progression.

## Discussion

TME is a dynamic system orchestrated by intercellular crosstalk that is responsible for tumor progression^3^. The exchange of cellular material between cells through various paracrine and endocrine mechanisms is an important pathway of intercellular crosstalk that is carried out via the secretion of exosomes^24^. Although diverse cancer cell-derived exosomes have been shown to be associated with the remodeling of the TME, the precise roles of these molecules are not fully understood^25^. Since CAFs are one of the most important cellular components in the TME^26^, and there is growing evidence suggesting that CAFs promote the progression of many cancers^27–29^, defining the mechanisms underlying the differentiation of NFs into CAFs has become critically important for developing novel cancer therapies. The clinical evidence in this study revealed that circulating exosomes from patients with CC can functionally affect fibroblast differentiation, suggesting that CC cell-derived exosomes influence stroma remodeling by regulating the activities of fibroblasts.

Wnt proteins have been reported to regulate cell differentiation and can act over a distance spanning many cells^16,17,30^. In this study, we observed that high Wnt2B expression correlated with an increased number of CAFs during cervical carcinogenesis, suggesting that CC cell-derived Wnt2B is associated with CAF conversion. However, it remains unclear how hydrophobic Wnt proteins travel in the extracellular space. Although it has previously been proposed that fat-body-derived lipoprotein particles are an extracellular vehicle for distributing Wnt proteins^31^, it is still not clear whether lipoprotein-bound Wnt proteins have any role in signaling activity^32^. Gross et al.^32^ reported that a substantial proportion of secreted Wnt proteins are found on exosomes, suggesting that exosomes may be ideal vehicles for their delivery and that Wnt proteins loaded into cancer-associated exosomes could be the versatile regulators of intercellular communication^16^. In this study, we found that serum exosomal Wnt2B levels were significantly higher in patients with CC than in healthy individuals. Moreover, our findings indicate that Wnt2B transfers directly from tumor cells to fibroblasts through exosomes, thereby upregulate the expression of α-SMA and FAP in vitro and in vivo, which is correlated with our initial clinical data. In addition to acquiring a CAF phenotype, the activated fibroblasts induced by exosomes with high Wnt2B levels gained enhanced proliferative and migrative properties. Thus, it is likely that exosomal Wnt2B initiates the CAF-phenotypic programming of cervical fibroblasts in the TME. Importantly, our study revealed that GW4869, an inhibitor of exosome secretion, led to intracellular Wnt2B accumulation and thus decreased Wnt2B secretion, while abrogating the activation of fibroblasts treated with CM containing high Wnt2B levels. Taken together, our results provide clear evidence that Wnt2B activates fibroblasts via exosomal transfer.

In addition to its induction, the maintenance of a CAF phenotype is also essential for the differentiation of NFs to CAFs^33^. Several studies have reported that activation of Wnt signaling in fibroblasts drives and maintains the phenotypic switch in fibroblasts^21,23^. Hamburg-Shields et al.^34^ found that sustained Wnt/β-catenin activity in dermal fibroblasts is related to fibrotic disease, and that the mechanism of Wnt2B action is dependent on the classic Wnt signaling pathway^32^. Our study has revealed that exosomal Wnt2B promotes nonP-β-catenin accumulation and translocation in the nuclear, where it engages DNA-bound TCF transcription factors. Widely used LEF/TCF reporters such as TOP-/FOP-flash system contain multimers of the TCF-binding motif, which represent Wnt signaling activity^35^. We further confirmed that exosomal Wnt2B significantly increased TOP-flash reporter activity compared with those in other groups, which can be abrogated using the Wnt/β-catenin signaling inhibitor HY-15597. Thus, our findings suggest that Wnt2B is expressed by exosomes secreted by CC cells in a form that is biologically active to drive Wnt/β-catenin signaling, and we confirmed that exosomal secretion is a critical route for the transfer of active Wnt2B. Exosomal Wnt2B secreted by CC cells not only initiated CAF-phenotypic changes in cervical fibroblasts residing in the TME but also triggered Wnt/β-catenin signaling in fibroblasts to sustain the CAF phenotype. Therefore, the transfer of Wnt2B from CC cells to fibroblasts via exosomes may be a crucial and efficient method of activating cellular functions involved in CAF conversion. Interestingly, these activated fibroblasts are in turn able to promote tumor growth and aggressiveness, thereby forming a positive feedback loop to strengthen the cancer progression, and providing evidence of CAFs activated by exosomal Wnt2B possessing tumor-promoting functions.

In conclusion, our results indicate that Wnt2B expression and circulating exosomal Wnt2B levels are often increased in CC cells. In addition, the horizontal transfer of exosomal Wnt2B secreted by CC cells into fibroblasts may promote the transition of NFs into CAFs by activating the Wnt/β-catenin pathway, which is capable of stroma remodeling and cancer progression. Thus, Wnt2B is a key factor that modulates the TME and may be a novel candidate for the diagnosis, prognosis, and targeted therapy of CC.

## Materials and methods

### Cell lines

The human CC cell lines SiHa, Caski, C33a, ME180, and Hela were purchased from the American Type Culture Collection (Manassas, VA, USA). HCerEpiC cells were purchased from ScienCell Research Laboratories (California, USA). Fibroblasts were extracted from cervical specimens. SiHa, Caski, C33a, and ME180 cells as well as fibroblasts were cultured in Dulbecco’s modified Eagle’s medium (Gibco, California, USA) supplemented with 10% heat-inactivated fetal bovine serum (Gibco). HeLa cells were cultured in Minimal Essential Medium (Gibco) supplemented with 10% heat-inactivated fetal bovine serum. HCerEpiC cells were cultured in the cervical epithelial cell medium (ScienCell Research Laboratories).

### Clinical specimens

All cervical specimens were obtained from patients at the Department of Gynaecological Oncology of The First Affiliated Hospital of Guangzhou Medical University and Nanfang Hospital (Guangzhou, China). This study was approved by the Institutional Research Ethics Committee. All patients provided written informed consent.

The paraffin-embedded cervical specimens included 11 normal cervical tissue samples, 17 cases of CIN I, 32 cases of CIN II–III, and 32 cases of CC. Of the fresh samples collected, 30 were from healthy individuals, 30 were cases of CIN I, 30 were cases of CIN II–III, and 30 were cases of CC. After the samples were excised, they were snap-frozen in liquid nitrogen and stored at −80 °C until further use.

### Primary cell extraction and purification

Tissue samples obtained under sterile conditions were transferred into petri dishes and washed five times with phosphate-buffered solution (PBS) containing 1% penicillin–streptomycin. The samples were then cut into pieces (~1 mm^3^) using ophthalmic scissors in a small amount of medium after scraping away epithelial cells and necrotic tissue. The tissue pieces were placed in a 25 cm^2^ cell culture flask and an appropriate amount of medium containing 10% fetal bovine serum (FBS) was added. According to the growth of the cells, the medium was changed every 2 days and a cell layer had overgrown the edge of the tissue within ~10 days. The tissue pieces were then removed from the flask, washed twice with PBS, and digested with 0.25% trypsin for 2 min. Once round and partially detached, the cells were immediately added to a medium containing 10% FBS to terminate digestion, transferred to a new cell culture flask, and incubated at 37 °C for 30 min. After incubation, some cells had attached and the medium was changed. When the cells had reached 80–90% confluency, the same purification steps were carried out a further three times to obtain pure fibroblasts.

### Exosome isolation and identification

Exosomes were isolated and identified from the cells as described by Zhou et al.^14^. Briefly, the exosomes were separated by mixing 10 mL of cell CM or 250 μL of serum with ExoQuick (California, USA) exosome precipitation solution, according to the manufacturer’s protocol. After incubation at 4 °C overnight, the mixture was centrifuged at 1500 × *g* for 30 min and the precipitated exosomes were subjected to electron microscopy, western blot, ELISA, and in vitro treatment. For TEM, the exosomes were fixed with 2% glutaraldehyde, stained with negative contrast phosphotungstic acid, and photographed by TEM (Hitachi, H-7500, Tokyo, Japan). For the western blot assays, exosomes were prepared using a Bicinchoninic Acid Protein Assay Kit (Beyotime, Shanghai, China). For in vitro treatment, 10 μg of exosomes were resuspended in 100 μL of PBS and added to 1 × 10^5^ recipient cells for 48 h. For ELISA, 250 μL of exosomes were resuspended in an equal amount of PBS.

### Immunofluorescence analysis

After the medium had been aspirated, cells were washed three times with PBS, fixed with 4% paraformaldehyde for 15 min at 25 °C, and permeabilized with 0.2% Triton X-100 for 10 min. After being washed a further three times, the cells were blocked with 10% FBS for 30 min at 25 °C, incubated overnight at 4 °C with primary antibodies, and then exposed to a fluorescent secondary antibody for 1 h at 25 °C. Cell nuclei were stained with DAPI (4′,6-diamidino-2-phenylindole) for 10 min at 25 °C and then observed under a fluorescence microscope (LSM 880, Carl Zeiss, Jena, Germany). Triple immunofluorescent staining was performed according to the manufacturer’s protocol (PerkinElmer Opal, Shanghai, China) and images were acquired using a confocal microscope (LSM 880, Carl Zeiss).

### Lentiviral transfection

We successfully constructed a lentiviral vector expressing CD63-GFP and transfected it into SiHa, Hela, and ME180 cells. The lenti-CD63-transfected cells were then transfected with mCherry-tagged lentiviral vectors expressing Wnt2B, silencing Wnt2B, or a negative control. Successful transfection was confirmed using confocal microscopy (LSM 880, Carl Zeiss) (Supplementary Fig. 1A), and the transfected cells were designated SiHa-Wnt2B, SiHa-shWnt2B, SiHa-NC, Hela-Wnt2B, Hela-shWnt2B, Hela-NC, ME180-NC, and ME180-Wnt2B.

### Statistical analysis

Statistical analysis was performed using SPSS V.20.0 software. Data are expressed as the mean ± standard deviation (SD). One-way analysis of variance was used for between-group comparisons. The *χ*^2^ test was used for categorical variables. Spearman’s rank test was used for correlation analysis. Differences were considered statistically significant at *p* < 0.05.

Detailed descriptions of the following techniques are available in the Supplementary Materials and Methods:

- Immunohistochemical analysis
- RNA extraction and qRT-PCR (primer sequences are shown in Table S2)
- Western blotting
- Wound healing assay
- Transwell migration assay
- Cell proliferation assay
- ELISA
- TOP-flash/FOP-flash reporter assay
- In vivo xenograft model

## Supplementary information

Supplementary Materials and Methods

Supplementary Figure and Table Legends

Supplementary Figure 1

Supplementary Figure 2

Supplementary Figure 3

Supplementary Figure 4

Supplementary Figure 5

Supplementary Figure 6

Supplementary Figure 7

Supplementary Tables

Email authorship confirmation

## Data Availability

The datasets used and/or analyzed during the current study are available from the corresponding author on reasonable request.
