# Supplementary material for: Tumor-secreted exosomal Wnt2B activates fibroblasts to promote cervical cancer progression
Source: Oncogenesis. 2021 Mar 17;10(3):30. doi: 10.1038/s41389-021-00319-w (PMC7969781; doi:10.1038/s41389-021-00319-w)
Supplement: Supplementary file 1 — Supplementary Materials and Methods [file 41389_2021_319_MOESM1_ESM.docx]

**Supplementary Materials and Methods**

***Immunohistochemical analysis***

The slides were incubated with primary antibodies against α-SMA (Abcam, Cambridge, UK, 1:200) and Vimentin (CST, Massachusetts, USA, 1:100) at 4 ℃ overnight, incubated with horseradish peroxidase-conjugated secondary antibodies (Abcam, Cambridge, UK, 1:1000), and then stained with diaminobenzidine colorimetric reagent solution (Zsbio , Beijing, China) and hematoxylin and eosin (Sigma Chemical Co. St.Louis, MO, USA). Since α-SMA staining is primarily observed in the cytoplasm and nucleus, we semiquantitatively evaluated tissue α-SMA expression by counting the positively stained areas in three random fields at ×100 magnification. The staining intensity score and percentage of immunoreactive cells were scored according to the following system: optical staining intensity was graded as negative (0), weak (1), moderate or medium (2), or strong (3); the proportion of positively stained cells was graded as 0, 0 %; 1, < 20 %; 2, 20–50 %; or 3, > 50 %. The final immunohistochemical score was obtained by multiplying the intensity score and the area score, with total scores of ≤ 3 classified as low expression and those of > 3 classified as high expression. Staining was evaluated independently by two blinded investigators.

***RNA extraction and qRT-PCR***

Total RNA was extracted from the cells and human CC tissues using TRIzol reagent (Invitrogen, California, USA) for qRT-PCR (ABI-7500, California, USA) according to the manufacturer’s protocol. The expression level of each mRNA was normalized to GAPDH as a reference gene and expressed as the fold difference compared to the control. Primer sequences are shown in Table S2.

***Western blotting***

Total protein was extracted from the cells and protein concentration determined using the BCA method (Beyotime, Shanghai, China). After denaturing at 100 ℃ for 5 min, 50 μg of each protein sample was separated by 10 % sodium dodecyl sulfate polyacrylamide gel electrophoresis at a constant pressure of 120 V and transferred onto PVDF membranes under a constant current of 200 mA. The membranes were blocked with 5 % skimmed milk for 60 min at 25℃ and incubated with the following primary antibodies overnight at 4 ℃: Wnt2B (Abcam, Cambridge, UK, 1:1000), α-SMA (Abcam, Cambridge, UK, 1:1000), FAP (Abcam, 1:1000), β-catenin (CST, Massachusetts, USA,1:1000), Non-phosphorylated (Active) β-catenin (Ser33/37/Thr41) (CST, Massachusetts, USA,1:1000), Vimentin (CST, Massachusetts, USA,1:1000), CD31 (Abcam, Cambridge, UK, 1:1000), and CK (Abcam, Cambridge, UK, 1:1000). GAPDH (Abcam, Cambridge, UK, 1:1000) was used as an internal control for semi-quantitative protein analysis. Next, the membrane was washed three times with PBS containing 0.1 % Tween-20 (PBST), incubated with HRP-conjugated secondary antibodies (Abcam, Cambridge, UK, 1:10000) for 1 h, and washed with PBST three times. Staining intensity was detected using an ECL chemiluminescent reagent (Pierce, Illinois, USA).

***Wound healing assay***

The cells were seeded in a six-well plate (4 × 10^5^/well) and cultured in DMEM containing 10 % FBS for one day. Once the cells had formed a monolayer, a wound was created in the plate using a sterile 10 μL pipette tip and the plate was washed with PBS three times to remove loose cells. After 0 and 24 h, the wounds were imaged using an inverted microscope at ×100 magnification (Olympus DP80, Tokyo, Japan) and Image J software was used to measure the healed wound area as a percentage of the total area to assess the migration of cells in the wounded area.

***Transwell migration assay***

Fibroblasts in different treatment groups were resuspended in DMEM without FBS and then added (150 μL; 5 × 10^4^ cells) to the upper Transwell chamber with a pore size of 8 μm, while 600 μL of medium containing 10 % FBS was added to the lower Transwell chamber. After 24 h, the chamber was removed, placed in 4 % PEA for 15 min, and dyed with crystal violet for 15 min. Finally, after a cotton swab had been used to clean the cells on the upper surface without moving them, the cells were washed with PBS three times, and the number of cells in ﬁve random ﬁelds counted under a microscope (×200; Olympus BX53).

***Cell proliferation assay***

Pretreated cells were seeded in 96-well plates (1.5 × 10^3^/well) and incubated in DMEM containing 10 % FBS at 37 ℃ and 5 % CO_2_. After 8 h (day 1), 100 μL of working solution (1:10) from a Cell Counting Kit 8 assay (Dojido, Japan) was added to each well for 2 h and the absorbance at a wavelength of 450 nm measured using a microplate reader. The absorbance was measured in the same way every 24 h until day 7 and a cell growth curve was drawn.

***ELISA***

Wnt2B levels in CM or cell lysates were quantified using a human Wnt2B ELISA Kit (cusabio, Wuhan, China) according to the manufacturer’s protocol. For the CM, 5 × 10^6^ cells were plated in a 10 cm dish with 15 ml of serum-free DMEM and the CM collected after 24 h. For the cells, 1 × 10^8^ cells were collected and stored at -20 ℃ overnight. After two freeze-thaw cycles to disrupt cell membranes, the cell lysates were centrifuged at 5000 × *g* for 5 min at 2-8 ℃ and the supernatant was collected.

***TOP-flash/FOP-flash reporter assay***

Cell transfection and luciferase assay according to the manufacturer's instructions, the NFs after different pretreatments were seeded into a 24-well plate in 0.5 mL cell culture medium at a density of 4×10^4^. After 12 hours, the medium was changed to a DMEM without fetal calf serum, and the TOP-flash reporter gene or FOP-flash control construct was transfected into the cells using Lipofectamine 2000 (Invitrogen, California, USA). The transfected cells were harvested 24 hours later. Luciferase activity was performed by using the luciferase reporter assay system (Promega).

***In vivo Xenograft model***

Female nude mice (4 weeks old) were purchased from the Laboratory Animal Center of Southern Medical University (Guangzhou, China). The studies were approved by the Institutional Animal Research Ethics Committee of Southern Medical University.

For fibroblasts activation *in vivo*[[1](#_ENREF_1)]: SiHa-Wnt2B, Hela-Wnt2B or ME180-Wnt2B (5.0×10^6^) were subcutaneously co-injected with NFs (5.0×10^6^) into the flank of nude mice. After tumor implantation, mice were intraperitoneally-injected with GW4869 (2.0mg/kg) or DMSO (solvent of GW4869, as a control), once every two days until three weeks. Then the primary tumors were harvested for IHC analysis.

For analysis of activated fibroblasts promote CC progression *in vivo*[[2](#_ENREF_2)]: SiHa, Hela or ME180 cells (5.0×10^6^) mixed with conditioned NFs (5.0×10^6^) pre-treated with exosomes derived from Siha-NC/Hela-NC/ME180-NC or Siha-Wnt2B/Hela-Wnt2B/ME180-Wnt2B were randomly injected into the right flank of nude mice (n=3 per group). Tumor size (mm3) was measured every 4 days and calculated by the formula: volume = (width)2 × length/2. The experiment was terminated when tumor size of the first mouse reached the maximum average diameter of 14 mm.

**References**

[1] Avgustinova A, et al. Tumour cell-derived Wnt7a recruits and activates fibroblasts to promote tumour aggressiveness. Nature communications. 2016;7:10305.

[2] Baroni S, et al. Exosome-mediated delivery of miR-9 induces cancer-associated fibroblast-like properties in human breast fibroblasts. Cell death & disease. 2016;7:e2312.
