# Supplementary material for: Tumor-secreted exosomal Wnt2B activates fibroblasts to promote cervical cancer progression
Source: Oncogenesis. 2021 Mar 17;10(3):30. doi: 10.1038/s41389-021-00319-w (PMC7969781; doi:10.1038/s41389-021-00319-w)
Supplement: Supplementary file 2 — Supplementary Figure and Table Legends [file 41389_2021_319_MOESM2_ESM.docx]

**Supplementary Figure and Table Legends**

**Supplementary Fig. 1 Cell identification. A.** Successful transfection was confirmed using confocal microscopy (×1,000): mCherry (red), CD63-GFP (green), and DAPI (blue). **B/D.** Western blot analysis for Wnt2B expression in the transfected cells. GAPDH was used as the loading control. **C/E.** Quantitative PCR analysis for Wnt2B expression in the transfected cells. Error bars represent the mean ± SD of three independent experiments. **P* < 0.05.

**Supplementary Fig. 2** **Wnt2B is highly enriched in exosomes secreted by ME180 cells. A.** Morphology of exosomes secreted by ME180 cells confirmed using transmission electron microscopy. Scale bar, 100 nm. **B.** Western blot analyses for positive markers (CD63 and CD81) and Wnt2B expression in exosomes secreted by ME180 cells. **C/D.** Western blot and ELISA analyses for Wnt2B in original CM from ME180-Wnt2B cells, or after overnight ultracentrifugation (160,000×*g*). **E/F.** Western blot and ELISA analyses for Wnt2B in the cell lysates and CM of ME180-Wnt2B cells treated with DMSO or GW4869 (15 μM) for two days. **B/C/E.** Exosomes or CM secreted by the same number of cells; GAPDH was used as the loading control. Error bars represent the mean ± SD of three independent experiments. **P* < 0.05.

**Supplementary Fig. 3 Identification of fibroblasts.** Western blot analysis of CD-31, CK, α-SMA, FAP, and Vimentin expression in human primary umbilical vein endothelial cells, SiHa cells, and primary NFs from normal cervical samples and CAFs from CC. Error bars represent the mean ± SD of three independent experiments. **P* < 0.05.

**Supplementary Fig. 4 Transport of exosomal Wnt2B. A.** NFs co-cultured with ME180-Wnt2B cells for 48 h imaged using confocal microscopy at ×630 magnification with morphology (BF), Wnt2B-mCherry (red), CD63-GFP (green), and DAPI (blue). **B.** Western blot analysis for Wnt2B in NFs cultured in the CM from ME180-Wnt2B cells treated with DMSO or GW4869 (15 μM), while NFs treated with DMSO or GW4869 in basal medium were used as the control group for analysis. **C.** ELISA analysis for Wnt2B in treated fibroblasts. **D.** Effect of treatment using exosomes derived from NFs and CAFs on the expression of Wnt2B in SiHa, Hela, and ME180 cells. **B/D.** GAPDH was used as the loading control. Error bars represent the mean ± SD of three independent experiments. **P* < 0.05.

**Supplementary Fig. 5 Exosomal Wnt2B secreted by tumor cells** **promotes fibroblasts activation. A.** Western blot analysis of α-SMA and FAP expression in NFs cultured alone (blank group) or co-cultured with the indicated exosomes (left); Western blot analysis for α-SMA and FAP expression in NFs pre-treated with CM from ME180-Wnt2B cells treated with DMSO or GW4869, while NFs treated with DMSO or GW4869 in basal medium were used as control groups for the analysis (right). **B/D.** Wound healing assay using treated fibroblasts. **C/E.** Migration assay using treated fibroblasts. **F/G.** Proliferation assay using treated fibroblasts. **H.** The role of exosomal Wnt2B on the activation of fibroblasts *in vivo* (×400). Error bars represent the mean ± SD of three independent experiments. **P* < 0.05.

**Supplementary Fig. 6** **Exosomal Wnt2B activates Wnt/β-catenin signaling in fibroblasts.** **A.** Cells stained for nonP-β-catenin (green) and DAPI (blue) for confocal microscopy analysis (×630). **B/ C.** The effect of Exo/CM secreted by tumor cells on the Top-flash activity of NFs**.**

**Supplementary Fig. 7** **Exosomal Wnt2B secreted by ME180 cells activates Wnt/β-catenin signaling in fibroblasts. A.** Western blot analysis of activation of Wnt/β-catenin signaling by total and nonP-β-catenin expression, and localization in NFs cultured alone (blank group) or co-cultured with the indicated exosomes (left). Western blot analysis of activation of Wnt/β-catenin signaling by total and nonP-β-catenin expression, and localization in NFs pre-treated with CM from ME180-Wnt2B cells treated with DMSO or GW4869, while NFs treated with DMSO or GW4869 were used as control groups for the analysis (right). GAPDH was used as a loading control for cytoplasmic proteins, and laminin B was used as a loading control for nuclear proteins. **B/ C.** Immunofluorescence analysis for nonP-β-catenin (green) localization in treated fibroblasts. Representative images are shown at ×630 magnification. D**.** The Top-flash activity of NFs with different treatment. **E.** Immunoblot assay results for CAF markers, total β-catenin and nonP-β-catenin, in fibroblasts treated with exosomes and incubated with DMSO or HY-15597 (10 μM) for one day.

**Table S1. Association between Wnt2B mRNA expression and clinicopathologic features during cervical carcinogenesis.**

**Table S2. Detailed primer sequences in the study.**
