# Supplementary figures and images for: Tumor-secreted exosomal Wnt2B activates fibroblasts to promote cervical cancer progression
Source: Oncogenesis. 2021 Mar 17;10(3):30. doi: 10.1038/s41389-021-00319-w (PMC7969781; doi:10.1038/s41389-021-00319-w)

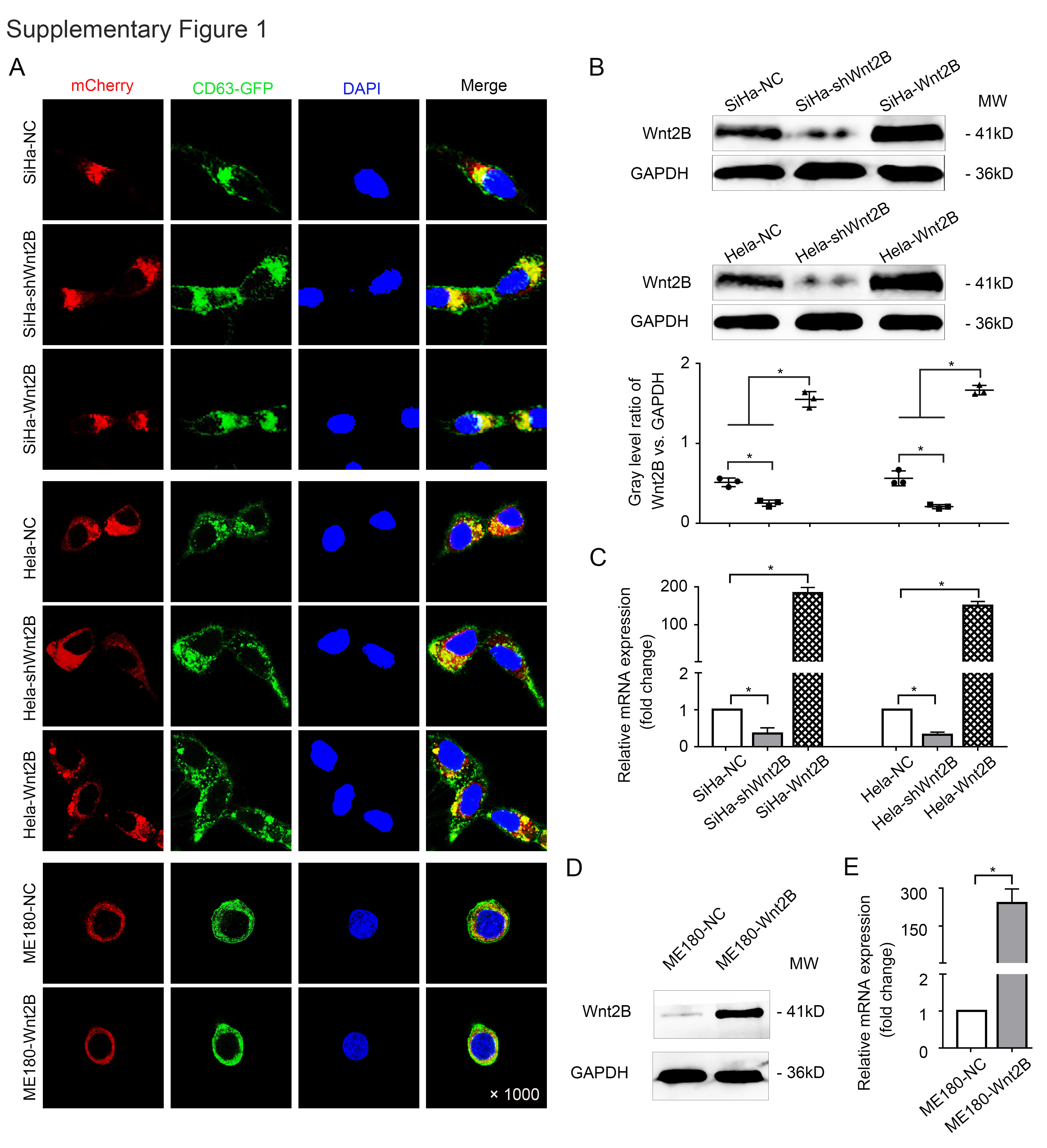

Supplement: Supplementary file 3 — Supplementary Figure 1 [file 41389_2021_319_MOESM3_ESM.tif]

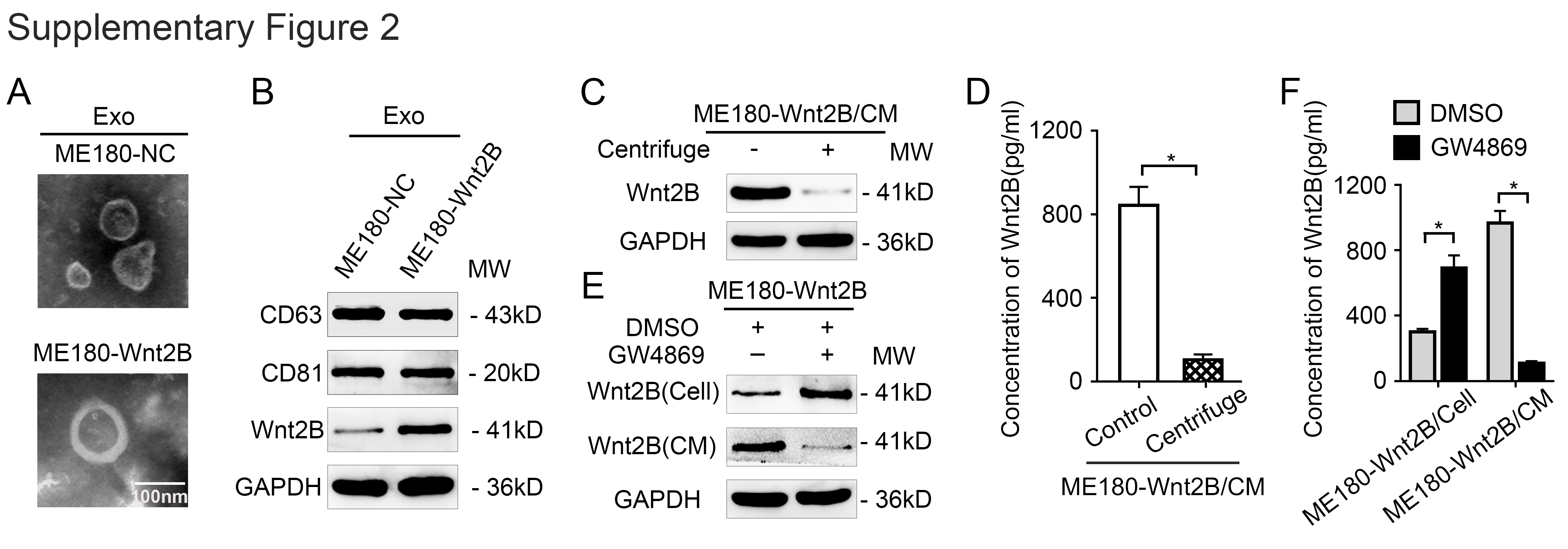

Supplement: Supplementary file 4 — Supplementary Figure 2 [file 41389_2021_319_MOESM4_ESM.tif]

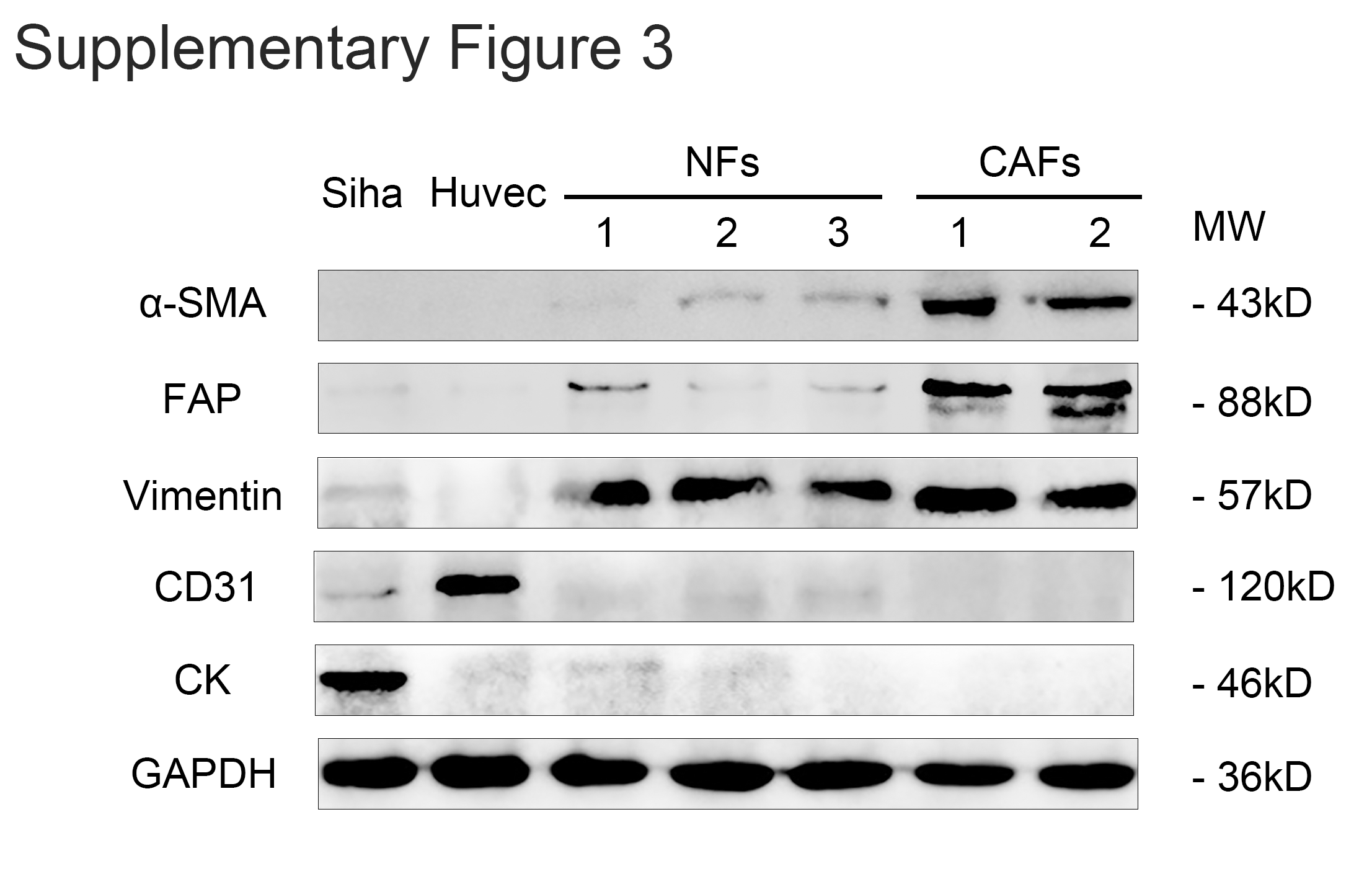

Supplement: Supplementary file 5 — Supplementary Figure 3 [file 41389_2021_319_MOESM5_ESM.tif]

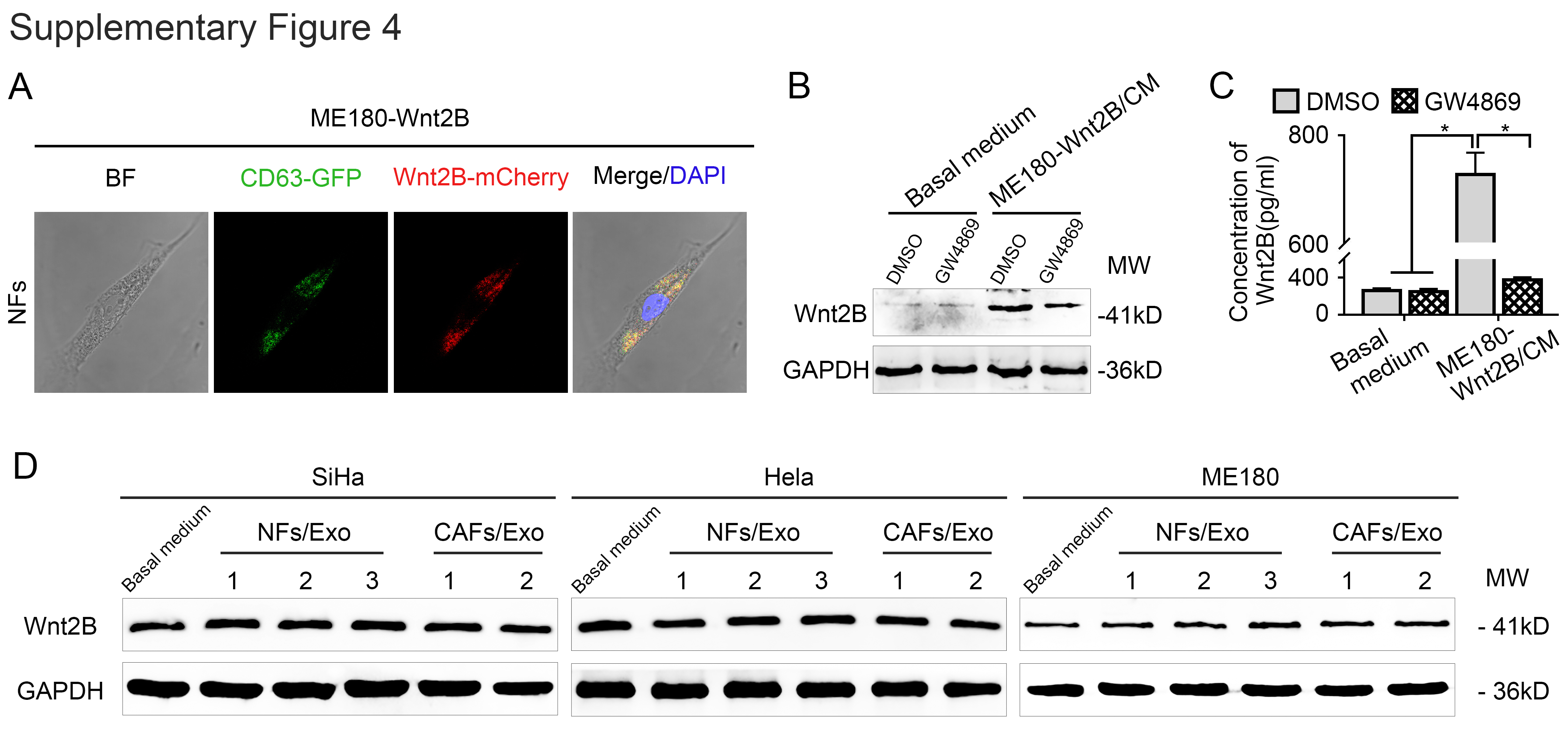

Supplement: Supplementary file 6 — Supplementary Figure 4 [file 41389_2021_319_MOESM6_ESM.tif]

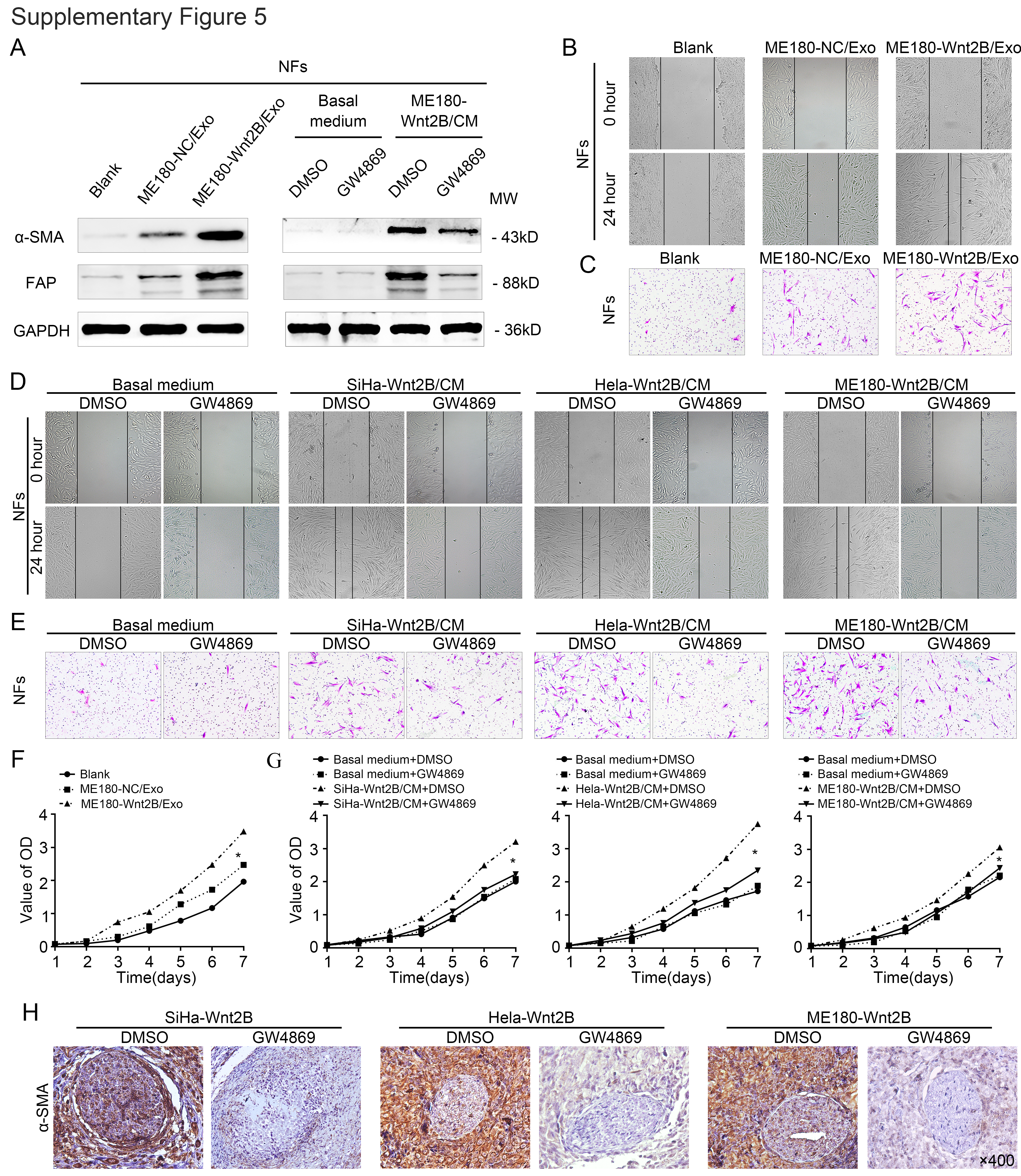

Supplement: Supplementary file 7 — Supplementary Figure 5 [file 41389_2021_319_MOESM7_ESM.tif]

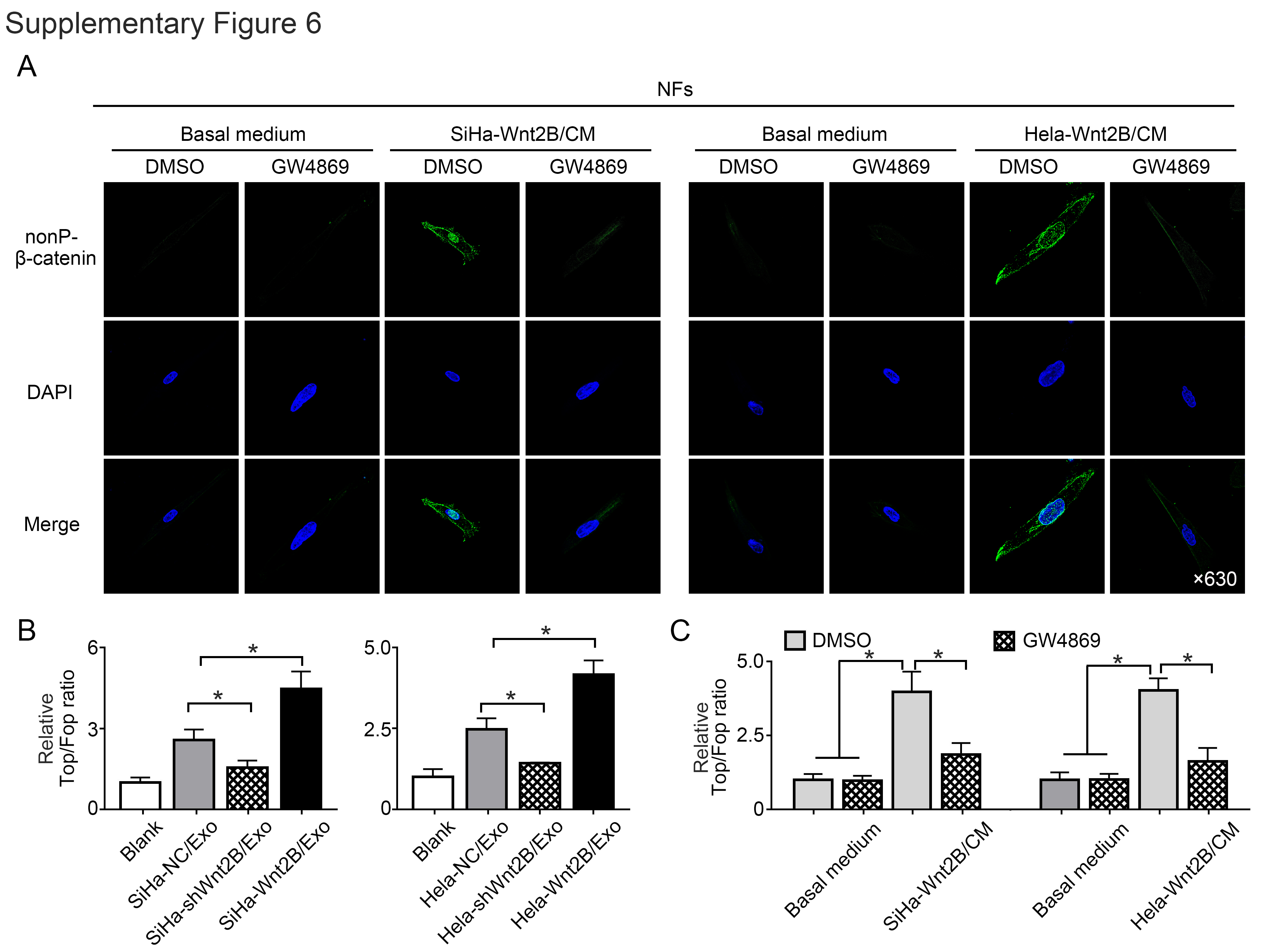

Supplement: Supplementary file 8 — Supplementary Figure 6 [file 41389_2021_319_MOESM8_ESM.tif]

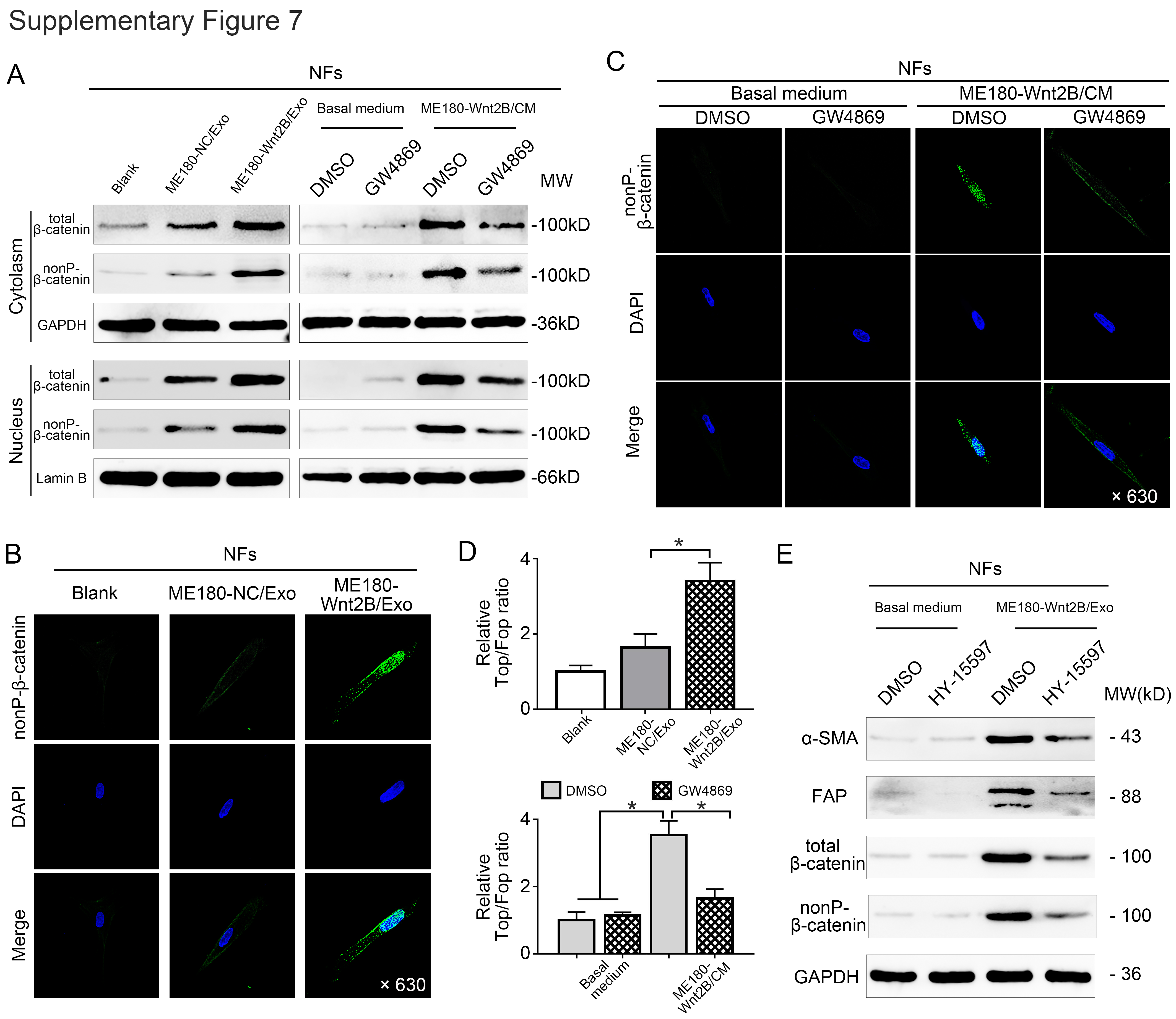

Supplement: Supplementary file 9 — Supplementary Figure 7 [file 41389_2021_319_MOESM9_ESM.tif]
