# Supplementary material for: Tumor-secreted exosomal Wnt2B activates fibroblasts to promote cervical cancer progression
Source: Oncogenesis. 2021 Mar 17;10(3):30. doi: 10.1038/s41389-021-00319-w (PMC7969781; doi:10.1038/s41389-021-00319-w)
Supplement: Supplementary file 10 — Supplementary Tables [file 41389_2021_319_MOESM10_ESM.docx]

**Table S1. Association between Wnt2B mRNA expression and clinicopathologic features during cervical carcinogenesis.**

| Variable | No. | mRNA expression of Wnt2B (fold change) | *p* |
| --- | --- | --- | --- |
| Group | 120 |  |  |
| Normal | 30 | 2.23±1.19 |  |
| CINI | 30 | 3.08±1.63 |  |
| CINII-III | 30 | 9.25±2.89 |  |
| CC | 30 | 15.39±3.58 | ＜0.01 |
| Age | 120 |  |  |
| ≤45 | 56 | 7.07±5.76 |  |
| >45 | 64 | 7.85±6.00 | 0.47 |
| HPV | 120 |  |  |
| + | 74 | 10.63±5.38 |  |
| - | 46 | 2.36±1.28 | ＜0.01 |
| Stage（CC） | 30 |  |  |
| I-IIA | 19 | 15.43±3.75  15.32±3.44 |  |
| IIB-IV | 11 |  | 0.94 |
| Histology（CC） | 30 |  |  |
| Squamous | 16 | 15.51±2.89 |  |
| adenoma | 14 | 15.26±4.35 | 0.85 |

Data are represented as mean ± SD; CIN = cervical intraepithelial neoplasia; CC = Cervical cancer; No. = number.

These clinical characteristics of patients related to Fig 1B and 1D.

**Table S2. Detailed primer sequences in the study.**

|  | Forward | Reverse |
| --- | --- | --- |
| Wnt1 | AGGTTCCATCGAATCCTGCAC | CATCTCGGAGAATACGGTCGT |
| Wnt2B | CGGGACCACACCGTCTTTG | GCGAGTAATAGCGTGGACTAC |
| Wnt3A | AGCTACCCGATCTGGTGGTC | CAAACTCGATGTCCTCGCTAC |
| Wnt4 | CTCCACACTCGACTCCTTGC | CCGAAGAGATGGCGTACACG |
| Wnt5B | CGCTTCGCCAAGGAGTTTG | TGCCATCTTATACACAGCCCT |
| Wnt7A | CTGTGGCTGCGACAAAGAGAA | GCCGTGGCACTTACATTCC |
| Wnt9A | CCACCGTGAGAAGAACTGC | GCCTGCACTCCACATAGCA |
| Wnt10B | GTGAGCGAGACCCCACTATG | CACTCTGTAACCTTGCACTCATC |
| GAPDH | ACAACTTTGGTATCGTGGAAGG | GCCATCACGCCACAGTTTC |
