# Supplementary material for: Tumor-secreted exosomal Wnt2B activates fibroblasts to promote cervical cancer progression
Source: Oncogenesis. 2021 Mar 17;10(3):30. doi: 10.1038/s41389-021-00319-w (PMC7969781; doi:10.1038/s41389-021-00319-w)

**All co-authors’ email responses are included as follow.**

**Contributing author # 1: Luojiao Liang**


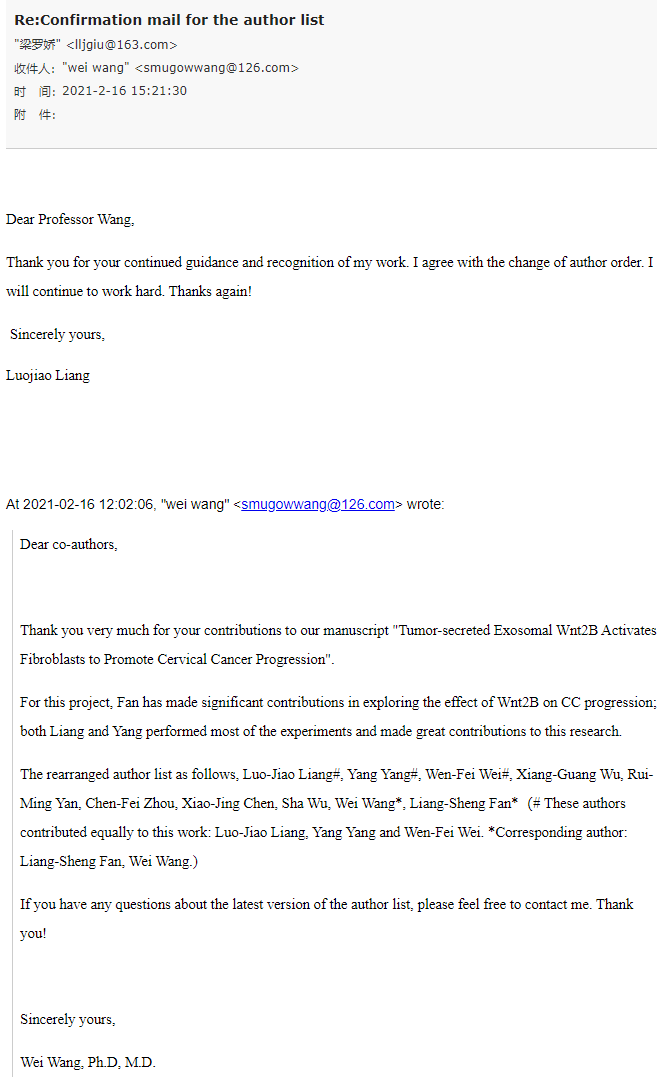


**Contributing author # 2: Yang Yang**


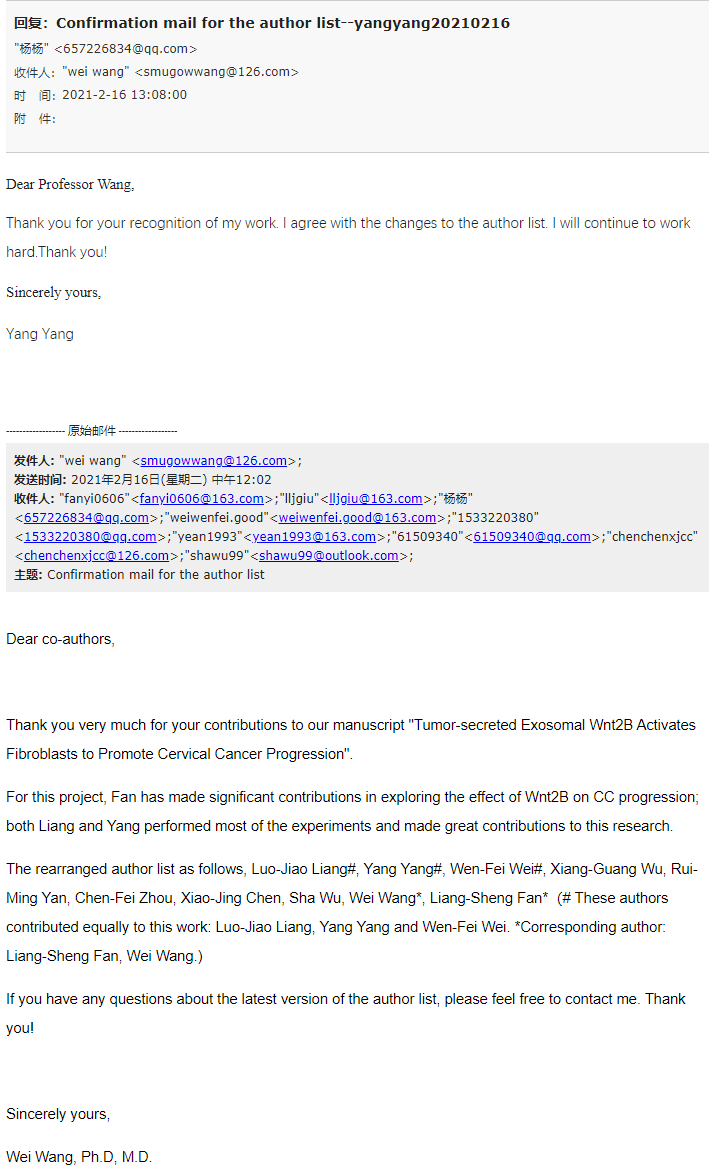


**Contributing author # 3: Wenfei Wei**


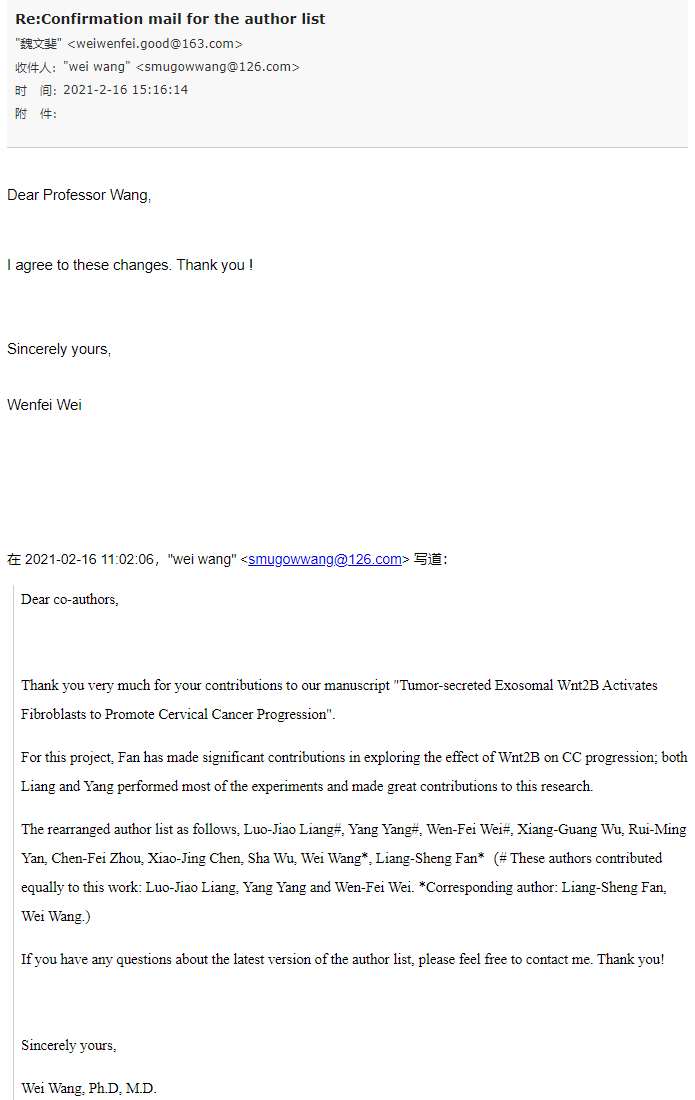


**Contributing author # 4: Xiangguang Wu**


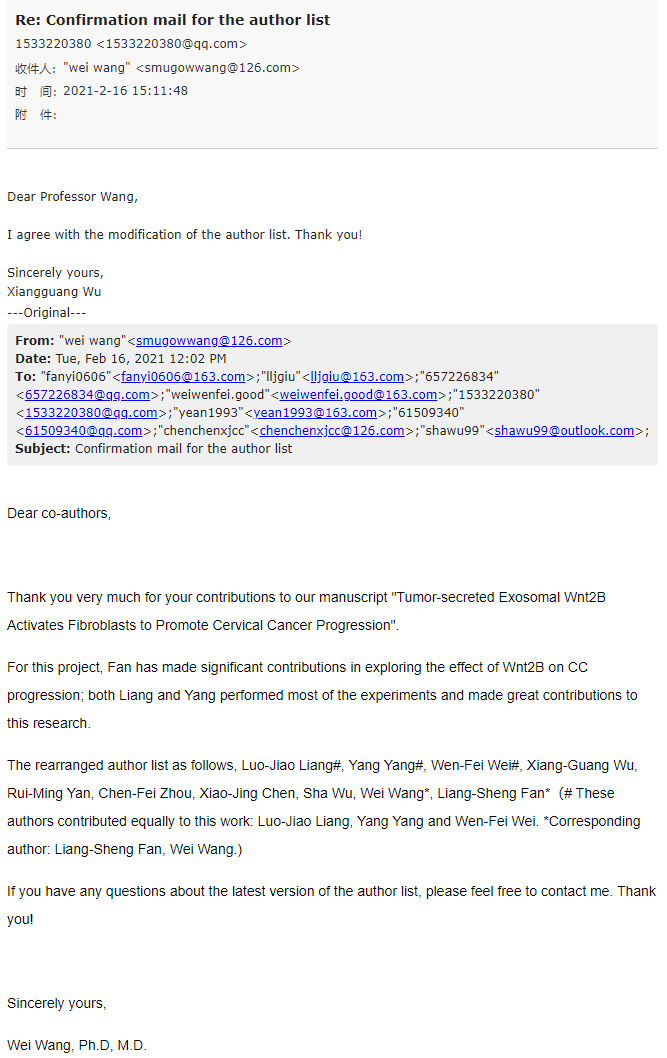


**Contributing author # 5: Ruiming Yan**


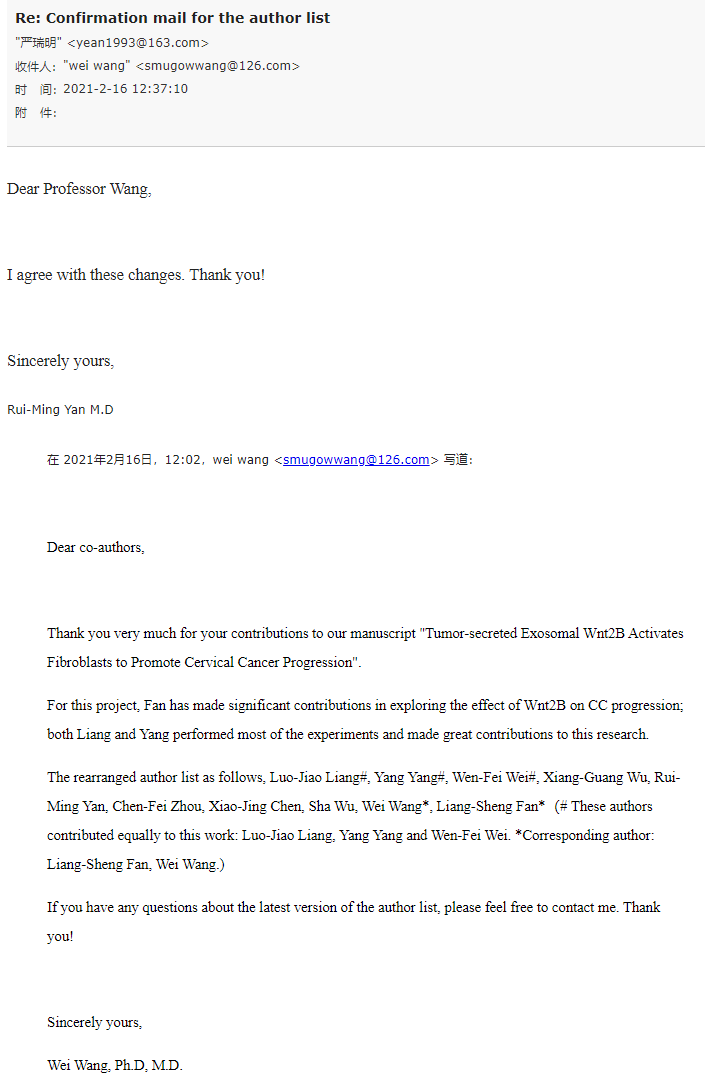


**Contributing author # 6: Chenfei Zhou**


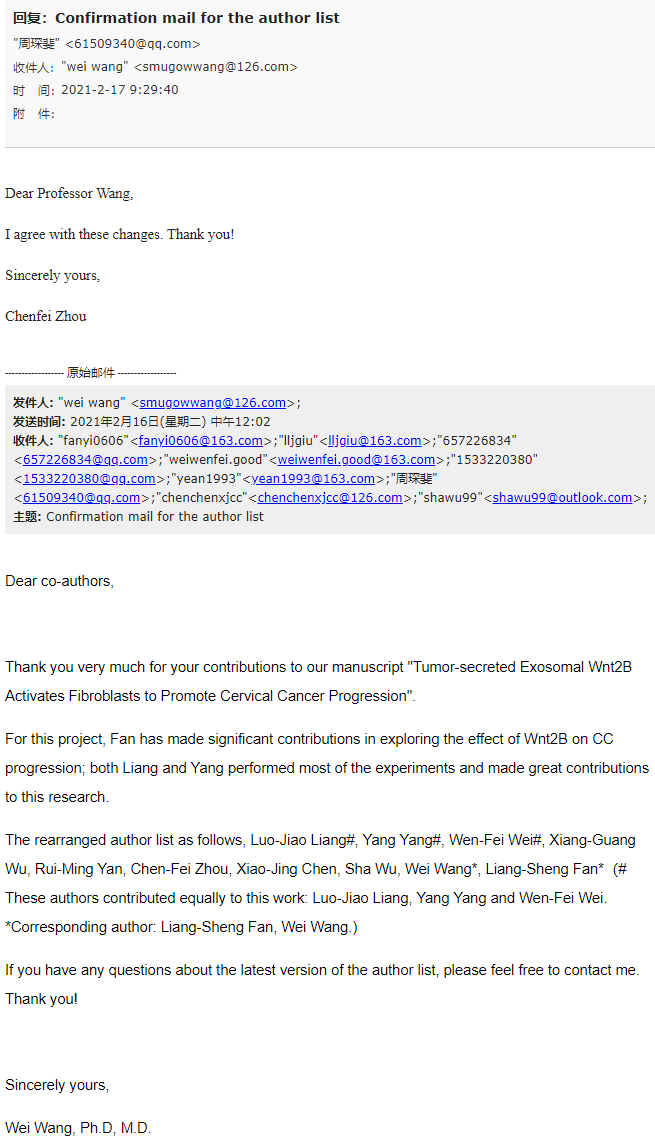


**Contributing author # 7: Xiaojing Chen**


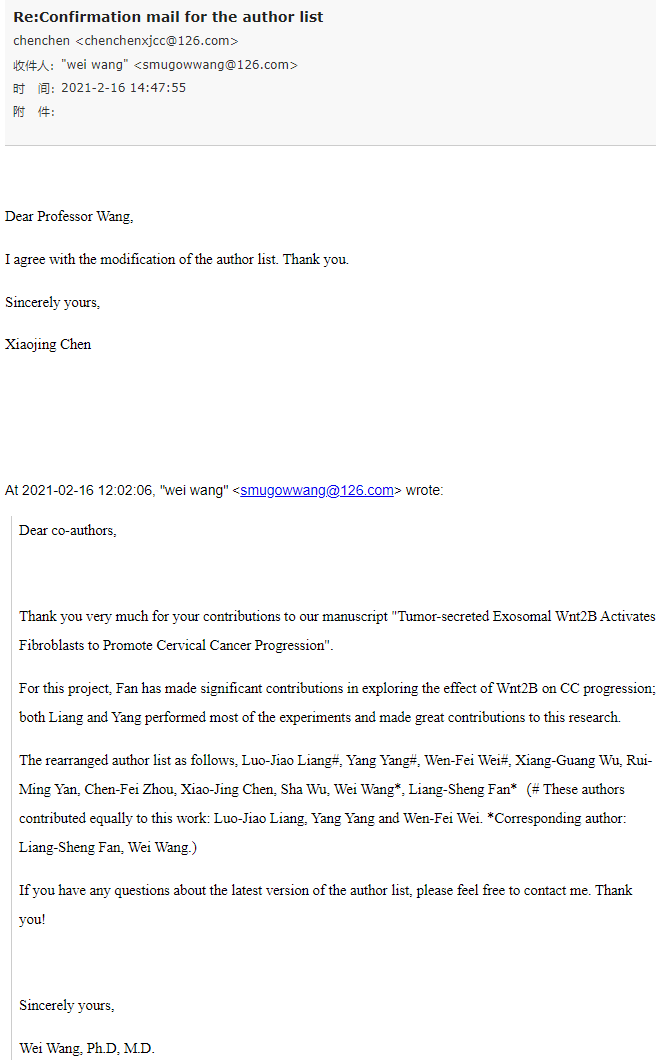


**Contributing author # 8: Wu Sha**


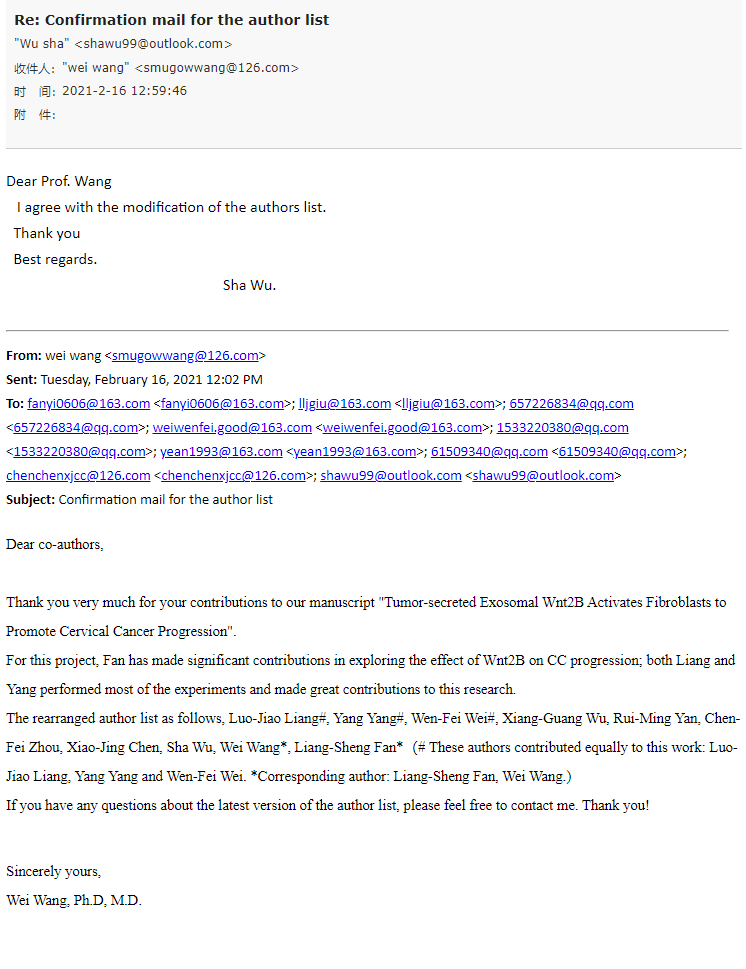


**Contributing author # 9: Liangsheng Fang**


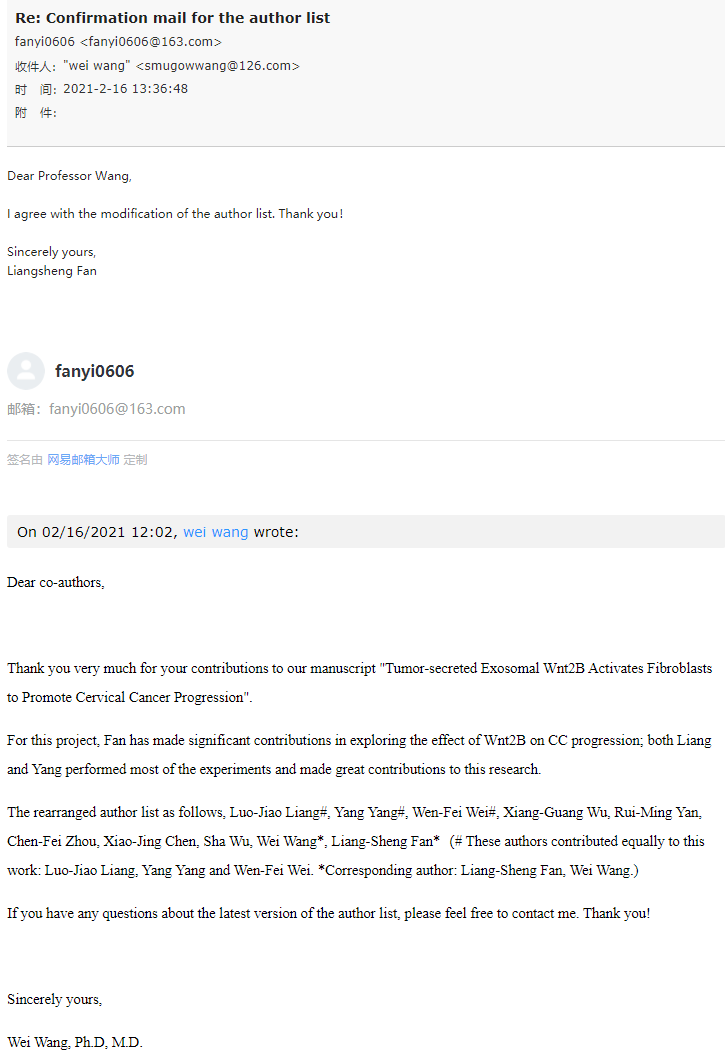


**The following document is a signed letter from all authors for the confirmation of the author list.**
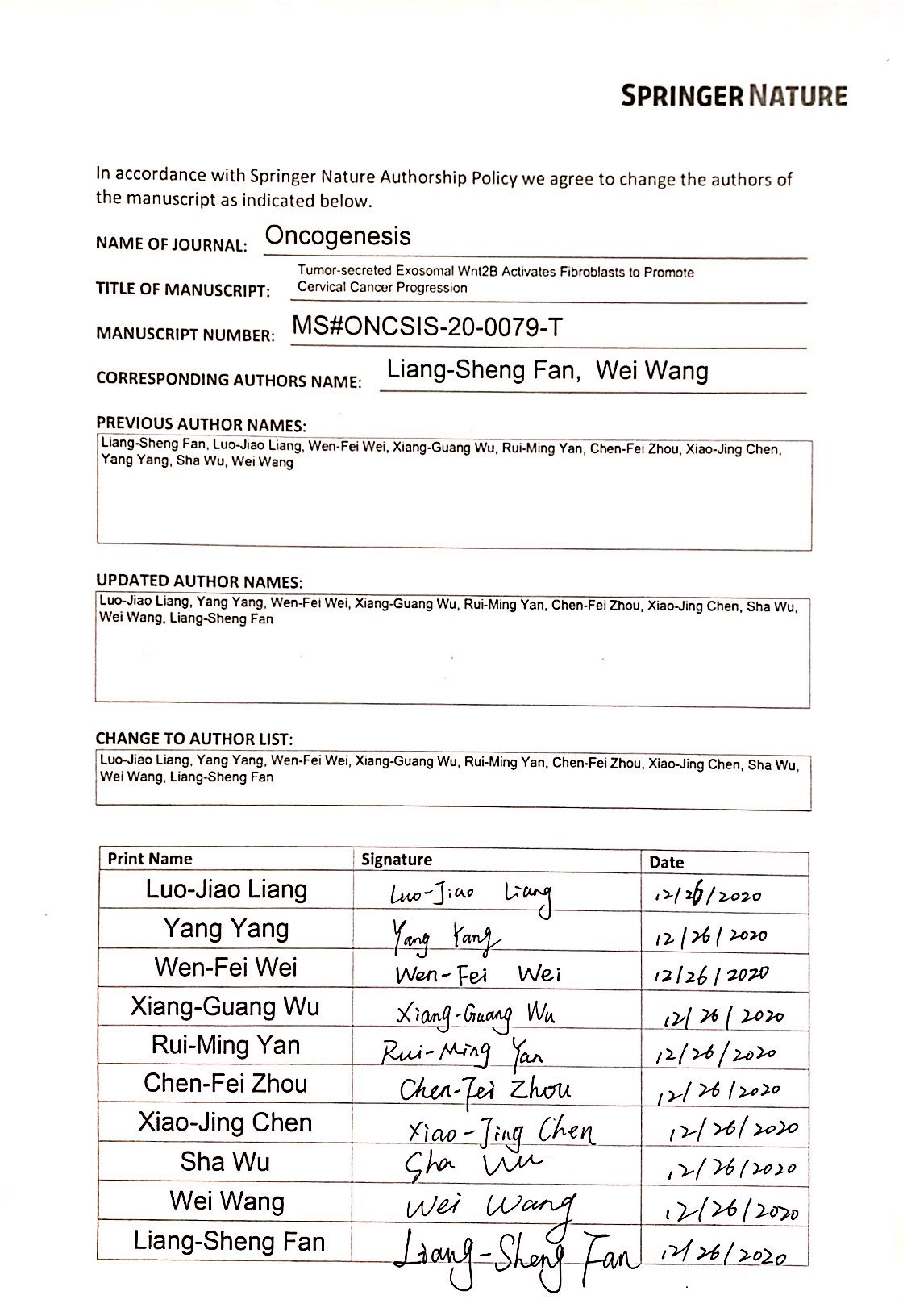

Supplement: Supplementary file 11 — Email authorship confirmation [file 41389_2021_319_MOESM11_ESM.docx]
